# Light Quantity Impacts Early Response to Cold and Cold Acclimation in Young Leaves of Arabidopsis

**DOI:** 10.1111/pce.15481

**Published:** 2025-03-27

**Authors:** Markéta Luklová, Marieke Dubois, Michaela Kameniarová, Klára Plačková, Jan Novák, Romana Kopecká, Michal Karady, Jaroslav Pavlů, Jan Skalák, Sunita Jindal, Ljiljana Tubić, Zainab Quddoos, Ondřej Novák, Dirk Inzé, Martin Černý

**Affiliations:** ^1^ Department of Molecular Biology and Radiobiology, Faculty of AgriSciences Mendel University in Brno Brno Czech Republic; ^2^ Department of Plant Biotechnology and Bioinformatics Ghent University Ghent Belgium; ^3^ VIB Center for Plant Systems Biology Ghent Belgium; ^4^ Laboratory of Growth Regulators, Institute of Experimental Botany The Czech Academy of Sciences & Palacký University Olomouc Czech Republic

**Keywords:** acclimation, freezing tolerance, jasmonic acid, leaf development, lipidome, proteome, transcriptome

## Abstract

Plant reactions to stress vary with development stage and fitness. This study assessed the relationship between light and chilling stress in Arabidopsis acclimation. By analysing the transcriptome and proteome responses of expanding leaves subjected to varying light intensity and cold, 2251 and 2064 early response genes and proteins were identified, respectively. Many of these represent as a yet unknown part of the early response to cold, illustrating a development‐dependent response to stress and duality in plant adaptations. While standard light promoted photosynthetic upregulation, plastid maintenance, and increased resilience, low light triggered a unique metabolic shift, prioritizing ribosome biogenesis and lipid metabolism and attenuating the expression of genes associated with plant immunity. The comparison of early response in young leaves with that in expanded ones showed striking differences, suggesting a sacrifice of expanded leaves to support young ones. Validations of selected DEGs in mutant background confirmed a role of HSP90‐1, transcription factor FLZ13, and Phospholipase A1 (PLIP) in response to cold, and the PLIP family emerged as crucial in promoting acclimation and freezing stress tolerance. The findings highlight the dynamic mechanisms that enable plants to adapt to challenging environments and pave the way for the development of genetically modified crops with enhanced freezing tolerance.

## Summary statement

*Arabidopsis thaliana* exhibits developmental‐stage‐dependent responses to light and cold stress, with early transcriptomic and proteomic changes revealing roles for HSP90‐1, FLZ13, and PLIP in acclimation. Young leaves prioritize plastid resilience, while expanded leaves show sacrificial responses.

## 1 Introduction

Given their sessile nature, plants are consistently exposed to a myriad of environmental stressors, seldom encountering singular abiotic factors (Kopecká et al. 2023). Recent studies have highlighted that simultaneous presence of environmental stresses triggers distinct molecular responses, transcending the mere aggregation of individual stress reactions (Zandalinas et al. 2020). The interplay between different signalling pathways can lead to a phenomenon known as acclimation. During evolution, plants developed this mechanism to increase their tolerance to abiotic stresses and to withstand the harsh conditions of the environment. The transcriptomic‐based induction of the acclimation can be delineated into several distinct stages. The initial stage, occurring within seconds, involves the rapid expression of genes to prevent irreversible damage. That is followed by the activation of early response genes, which lay the foundation for long‐term protection. Next, late response genes are triggered, initiating systemic acclimation mechanisms. Finally, the de‐acclimation process prepares the plant for stress resolution. A disruption in any of the acclimation steps may compromise the plant's ability to adapt and significantly impact its resilience (Zandalinas et al. 2019).

The plant's reaction varies based on its stage of development and overall fitness (Peck and Mittler 2020). For instance, when exposed to high‐light stress, young leaves activate protective mechanisms like non‐photochemical quenching, which might not be present or as effective in mature leaves (Rankenberg et al. 2021). Moreover, when subjected to stress, younger leaves tend to show increased levels of anthocyanin and possess a higher ability to remove reactive oxygen species (Zhu et al. 2018). Mature leaves seem to undergo a reduction in their ability to start the detoxification process, resulting in decreased tolerance to photoinhibition. The phenomenon of young leaves being prioritized for recovery has been found across various abiotic stresses. For instance, during drought stress, the accumulation of abscisic acid (ABA) initiates a process leading to the transport of carbohydrates and early senescence in mature leaves. Simultaneously, this stimulus suppresses growth and promotes the absorption of nutrients by young leaves (Sperdouli and Moustakas 2014; Schippers et al. 2015; Zhao et al. 2016).

In temperate regions, sudden temperature drops and freezing stress pose significant threats to plant survival and agricultural production (Kopecká et al. 2023). Cold stress disrupts various physiological processes essential for plant growth and survival, including membrane fluidity, nutrient uptake, and energy production. Despite our knowledge of integral components of cold perception pathway, the exact mechanism has not been identified (Kerbler and Wigge 2023). It is believed that plasma membrane is the primary source of signalling and several membrane‐associated proteins have been proposed as candidate thermosensors, including ANNEXIN1 that mediates cold‐triggered Ca^2+^ influx and freezing tolerance in *Arabidopsis thaliana* (Wei et al. 2021), protein COLD1 that mediates chilling tolerance in *Oryza sativa* through G‐protein signalling (Ma et al. 2015), and calcium/calmodulin‐regulated receptor‐like kinase that modulates cold acclimation through MAP kinase cascade (Yang et al. 2010). Substantial evidence also implicates a role of the circadian clock components ELF3, LHY, and PPR7 (Jung et al. 2020; Wu et al. 2024; Kim, Kim, and Somers 2024) and light perception pathway, including Phytochrome B, phytochrome‐interacting factors, and interactions with chromatin (reviewed in Kerbler and Wigge 2023).

Following the first exposure to cold shock, rapid alterations in the plasma membrane are initiated, when diacylglycerol kinase (DGK) undergoes activation after exposure to cold temperatures, leading to the conversion of diacylglycerol (DAG) into phosphatidic acid (PA) (Arisz et al. 2013). This enzymatic reaction is followed by alterations in the membrane fluidity, leading to the activation of many second messengers, including ROS, inositol phosphates, and calcium ions, that are recognized by specific protein sensors (Wei et al. 2021). The MAP kinase cascade facilitates the regulatory phosphorylation of downstream signalling components in response to cold stress. In *Arabidopsis*, the activation of the cascade is initiated by mitogen‐activated protein kinase kinase kinase (ANP1) and leads to positive regulation of freezing tolerance (Zhang and Sonnewald 2017). The MAP kinase cascade is also responsible for regulating the activity of ROS‐scavenging enzymes to maintain redox equilibrium during periods of cold stress. All these mechanisms lead to the activation of the main established cascade, ICE‐CBF (Inducer of CBF expression, C‐repeat/dehydration‐responsive element‐binding factor) (Chinnusamy, Zhu, and Zhu 2007; D.Z. Wang, Jin, et al. 2017; Hwarari et al. 2022). Multiple positive and negative regulators have been found for various transcription factors involved in cold response. These regulators include calmodulin‐responsive transcriptional 3 (CAMTA3, At2g22300; Doherty et al. 2009), open stomata 1 (OST1, AT4G33950; Ding et al. 2015), E3 ubiquitin‐protein ligase HOS1 (HOS1, At2g39810; Ishitani et al. 1998), E3 SUMO‐protein ligase SIZ1 and 2 (SIZ1; Miura and Ohta 2010), inducer of CBF expression 1 (ICE1, At3g26744; Chinnusamy et al. 2003), Transcription factor MYB15 (MYB15, At3g23250; Agarwal et al. 2006) and STRUBBELIG‐receptor family 6 (SRF6, At1g53730; Knight and Knight 2000).

The whole mechanism is quite complex, with more than 3000 identified cold‐responsive genes and overlaying regulator circuits that integrate inputs from other signalling pathways, including that of phytohormones and light. For instance, positive regulator of ethylene signalling EIN3 exerts a negative regulatory effect on the expression of CBFs (Shi et al. 2012), and inhibitors of the jasmonic acid signalling (JAZ1 and JAZ4) interact with ICE1 and ICE2 transcription factors inhibiting their activity (Yang et al. 2019). An additional part of the regulatory circuit is thioredoxinTRX‐H2, which reduces CBFs in the nucleus, ultimately activating COR genes (Lee et al. 2021).

In plants evolutionarily adapted to cold, the cold response is likely to result in cold acclimation. Cold acclimation represents a complex physiological and molecular response to low temperatures, involving dynamic changes in gene expression, protein abundance, and metabolite composition that culminate in a new homeostatic state (Liu et al. 2024; John et al. 2025). It is frequently linked to improved freezing tolerance (Kerbler and Wigge 2023; Nagel et al. 2024; Kosová et al. 2025) and plant survival represents a more comprehensive measure of its success (Hincha and Zuther 2020). However, cold acclimation extends beyond freezing resistance, as certain physiological adaptations, such as modifications in photosynthesis, do not necessarily confer increased tolerance to freezing temperatures (Goldstein et al. 1996). Rather than a singular response, cold acclimation orchestrates a suite of partially independent processes that collectively contribute to establishing a new equilibrium under low‐temperature conditions. These processes help maintain basal cellular functions, sustain energy‐demanding defence mechanisms, and prime the plant for exposure to more extreme temperatures. At subzero temperatures, ice crystal formation poses a major challenge, disrupting water potential and causing mechanical damage through crystal expansion. To mitigate these effects, plants employ diverse protective strategies, including antifreeze proteins that inhibit ice nucleation and recrystallisation (Nagel et al. 2024), late embryogenesis abundant (LEA) proteins that stabilize proteins and membranes (Kosová et al. 2021), and cold‐induced metabolites that contribute to osmotic balance, reactive oxygen species scavenging, and other defence mechanisms (Nagel et al. 2024; Kosová et al. 2025).

Light quality and quantity is critical for cold acclimation, as evidenced in recent publications (Prerostova et al. 2021; Novák et al. 2021; Kameniarová et al. 2022; Sugita et al. 2024). Mutants in *PHYB* showed upregulated CBF expression (Jiang et al. 2020) and modulated circadian clock and ROS metabolism (Luklová et al. 2022). In our previous work, we established a model experiment that allowed us to follow the impact of light intensity on freezing resilience (Novák et al. 2021). We showed that the acclimation under lower photosynthetic photon flux density (PPFD) has a contrasting mechanism to that found under standard PPFD. In our previous research, analysing the entire plant led to a notable bias. The interpretation was predominantly based on the molecular profile of fully expanded leaves, which constitute the bulk of the plant's biomass. Here, we present a more detailed analysis focused on young leaves, the tissue known to be actively protected by the plant. Consequently, this tissue is expected to employ distinct mechanisms compared to those found in older leaves, and provide more insight into the mechanisms behind cold resilience.

## 2 Materials and Methods

### 2.1 Plant Material and Growth Conditions

To study the effect of light intensity on cold stress, we have employed the plant model *Arabidopsis thaliana* (L.) Col‐0. Seeds were surface sterilized by immersion in 75% and 96% ethanol, respectively, and stratified in water for 3 days (4°C, dark conditions). Plants were cultivated in AR‐36L growth chambers (Percival Scientific Inc, Perry, IA, USA) under short‐day photoperiod (65% relative humidity; 21/19°C day/night temperatures, photon flux density (PPFD) 100 µmol m^−2^ s^−1^ provided by fluorescent tubes Philips TL‐D) using an Araponics hydroponic system (Araponics, Liege Belgium, 1.7 l tank) in a half‐strength Murashige and Skoog media. Growth media was refreshed every 7 days. Plants reaching the growth stages L(1.06) ‐ (6 rosette leaves are greater than 1 mm) and L(1.14) were divided into four sub‐groups (*n* > 42) and cultivated at the following conditions: (i) S‐PPFD at (100 µmol m^−2^ s^−1^), 21°C (S); (ii) low‐PPFD (at 20 µmol m^−2^ s^−1^; LL), 21°C; (iii) S‐PPFD at 4°C (C) and (iv) low‐PPFD, 4°C (CLL). Ten leaves n.6 L(1.06) and 10 expanded leaves n.6 L(1.14) from three biological replicates (transcriptome, proteome of expanded leaves) were harvested after 3 h of treatment. For the L(1.06) proteome, one leaf n.6 was collected from each of a subset of ten biological replicates. Leaves were flash‐frozen in liquid nitrogen, homogenized, and aliquoted for molecular analyses.

Plants for determination of lipid and hormone content of selected mutant lines—*plip2‐1*, *plip2‐2*, *plip3‐1*, *plip3‐2, plip1,2,3* line 1, *plip1,2,3* line 2 and overexpression lines *PLIP2*‐OX line 1, *PLIP2*‐OX line 2 and *PLIP3*‐OX line 2 (kindly provided by Prof. Christopher Benning, MSU) were prepared as follows: plants were cultivated on Petri plates for 2 weeks period at short day conditions (8 h/16 h light/dark). After this period, Petri plates were transferred into the following conditions: (i) S (ii) LL, (iii) C and (iv) CLL. The samples were collected for hormone and lipid analyses after 3 and 147 h of exposure to the treatment. For phytohormone analyses, five biological replicates, each pooled from at least 60 plants, were collected. For lipidome analyses, at least three biological replicates, each consisting of 25 plants, were collected. Plant material was snap‐frozen in liquid nitrogen and stored at −80°C until further use.

Freezing survival assays were performed as follows: Arabidopsis Col‐0 and mutant lines were cultivated on ½ Murashige‐Skoog medium supplemented with 0.8% agar in 8/16 (day to night) day‐length at S conditions. After 2 weeks of cultivation on horizontal agar plates, plantlets were cold acclimated using C and CLL conditions for 7 days. The freezing survival assay was performed using the adjusted method by (Perea‐Resa, Catalá, and Salinas 2020) with following changes: after a week of C‐ and CLL‐acclimation, plants were exposed to decreasing temperature at rate 2°C per hour until the temperature in the growth chamber reached −6°C. At this point, the ice nucleation was induced. Next, plants were exposed to programmed cycle of temperature reduction—decrease of 1°C per 2 h. Selected temperatures (−7°C, −8°C, −9°C, −10°C, −11°C and −12°C) were maintained for 2 h, and Petri plates were then allowed to thaw at 4°C. After 14 days of recovery at S, the survival rates were calculated by counting the number of plants that produced new leaves. For each line, PPFD, and temperature point, at least three biological replicates, each consisting of at least 25 plants, were analysed. To estimate LT_50_ the R package MASS was utilized (Venables and Ripley 2002). A binomial logistic regression model was fitted with temperature as the predictor and mortality percentage as the response variable. The dose.p() function was used to estimate LT_50_, while the confint() function provided a confidence interval, quantifying the uncertainty around the estimate.

For the chloroplast analysis, plantlets were cultivated at 21°C under S‐PPFD for 2 weeks. The young developing leaves were observed with a confocal laser scanning microscope LSM700 (Carl Zeiss, Germany) equipped with a Plan‐Apochromat 40× objective and an argon‐neon laser with a wavelength 488 nm. Palisade mesophyll cells were scanned in five leaf regions with 448 × 362 frame (scaling per pixel: 0.2418 µm × 2.2418 µm × 0.25 µm). Image post‐processing was performed using ZEN software (Carl Zeiss, Germany). Chloroplast volume and number of chloroplasts per area were determined using the Z‐stacks and 3D Object Counter in ImageJ 1.54d (Schneider, Rasband, and Eliceiri 2012). The experiment was done in three biological replicates, each consisting of five plants.

### 2.2 RNA Sequencing and Differential Expression Analysis

The sequencing was performed at the Nucleomic Core facility (VIB, Leuven, Belgium, www.nucleomics.be). Samples were sequenced using single‐end mode with a read 700 nt bp long using Illumina NextSeq500. Quality control was performed by using the Galaxy platform with FastQC; alignment was performed with the Salmon algorithm (Patro et al. 2017). Sequences were searched against the Arabidopsis reference genome ARAPORT11. Differential gene expression (DEG) was determined for uniquely expressed genes with the EdgeR package (Robinson, McCarthy, and Smyth 2009) against control treatment (S). Genes were determined to be differentially expressed with a false discovery rate (FDR) adjusted FDR ≤ 0.05. Visualisation and statistical analyses were performed with use or R packages: pheatmap (Kolde; 2015), Bioconductor (Gentleman et al. 2004), ggplot2 (Wickham 2011), R software (v.4.0.3) (R Core Team, 2020, R Foundation for Statistical Computing, Vienna, Austria).

### 2.3 Proteomic Analyses

Total protein extracts were prepared as described previously (Dufková et al. 2023). Portions of samples corresponding to 5 µg of peptide were analysed by nanoflow reverse‐phase liquid chromatography–mass spectrometry using a 15 cm C18 Zorbax column (Agilent), a Dionex Ultimate 3000 RSLC nano‐UPLC system and the Orbitrap Fusion Lumos Tribrid Mass Spectrometer (Thermo). Peptides were eluted with up to a 120‐min, 4%–40% acetonitrile gradient. Spectra were acquired using the default settings for peptide identification, employing HCD activation, resolution 60 000 (MS) and 15 000 (MS2), and 60 s dynamic exclusion. The measured spectra were recalibrated and searched against the Araport 11 protein database. Only proteins with at least two unique peptides were considered for the quantitative analysis. The quantitative differences were determined by Minora, employing precursor ion quantification followed by normalisation. The mass spectrometry proteomics data have been deposited to the ProteomeXchange Consortium via the PRIDE (Perez‐Riverol et al. 2022) partner repository with the data set identifier PXD050271.

### 2.4 Lipidomic Analyses

Total lipids were extracted as described previously (Dufková et al. 2023). In brief, 30 mg of plant material was extracted in tert‐butyl‐methyl‐ether:methanol mixture. The nonpolar fraction was separated and 200 µL aliquots were dried by vacuum centrifugation, resolved in 200 µL of isopropanol/methanol/tert‐butyl‐methyl‐ether 4/2/1 supplemented with 20 mM ammonium formate and analysed by direct infusion using Triversa Nanomate (Advion Biosciences) nanoelectrospray source and the Orbitrap Fusion Lumos Mass Spectrometer (Thermo Scientific). Obtained spectra were analysed by the software Freestyle 1.7 and LipidSearch 4.2 (Thermo Scientific).

### 2.5 Phytohormone Analyses

Quantification of phytohormones and related compounds was performed using liquid chromatography‐tandem mass spectrometry (LC‐MS/MS) following the methodology by Karady et al. (2024). cisOPDA was quantified according to Široká et al. (2022). For all compounds, concentrations were assessed using the standard isotope dilution method. All used solvents were of analytical or higher grade (Merck/Sigma‐Aldrich KGaA, Darmstadt, Germany).

### 2.6 Statistics

For evaluating statistical significance, Student's *t*‐test, Fisher's exact test, chi‐squared test, the Kruskal–Wallis test, one‐way and two‐way ANOVA, and edgeR were used. Statistical tests were implemented using R, MetaboAnalyst (Pang et al. 2021), and the Real Statistics Resource Pack software for MS Excel (Release 6.8; Copyright 2013–2020; Charles Zaiontz; www.real-statistics.com). The reported statistical tests were generated and implemented using default and recommended settings unless otherwise indicated. Significant differences refer to *p* < 0.05 and adjusted adj. *p* < 0.05 (the Benjamini‐Hochberg procedure, 5% FDR).

## 3 Results

### 3.1 Light Intensity Impacts Cold Acclimation and Freezing Stress Resilience

**Figure 1:**
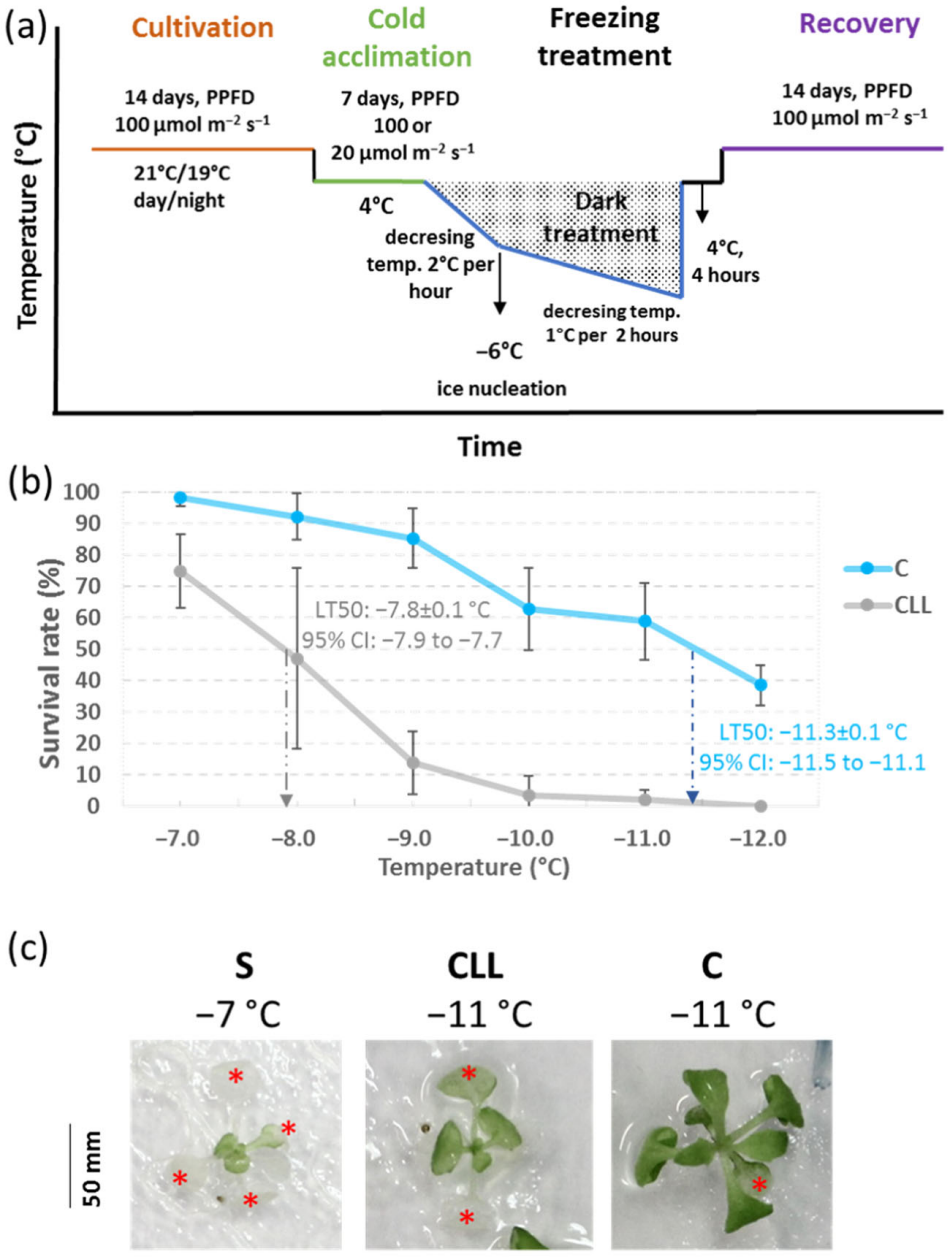
Freezing resistance in Arabidopsis plants. (a) Experimental design. (b) Freezing survival monitoring after 2 weeks of recovery period. The plot represents the means and standard deviation (three biological replicates, *n* > 25); arrows mark calculated LT_50_. (c) The impact of freezing stress on leaves. Representative images demonstrating the promoted survival of younger leaves documented in the survival assay experiments. *Arabidopsis thaliana* Col‐0 plants were acclimated under indicated conditions (S—100 µmol m^−2^ s^−1^, 21°C; C—100 µmol m^−2^ s^−1^ 4°C; CLL—20 µmol m^−2^ s^−1^, 4°C) and exposed to freezing stress for 2 h. The images were taken after 2 weeks of recovery; asterisks (*) indicate chlorotic tissues.

Our previous work showed that both quality and quantity of light impact response to cold stress (Novák et al. 2021; Kameniarová et al. 2022). Here, to validate the previous observations made under artificial conditions of hydroponic culture, Arabidopsis plants (Col‐0) were grown as described in materials and methods, and the response to freezing was monitored by calculating LT_50_ using a survival assay. Our previous analyses targeted conditions that resulted in approximately 50% mortality rates of control plants. Here, a more in‐depth temperature profile was analysed, and freezing was applied (Figure 1a). The comparison of survival rates between standard PPFD (C) and low PPFD (CLL) at 4°C showed statistically significant differences (*p* < 0.001) with corresponding LT_50_ of −11.3 ± 0.1°C and −7.8 ± 0.1°C for C and CLL, respectively (Figure 1b). Besides the differences in the LT_50_ values, our observations indicated a development‐dependent response to cold that seemed to promote the survival of younger leaves (Figure 1c).

### 3.2 Young Leaf Transcriptome in Early Response to Cold Showed Significant Impact of Light Intensity

**Figure 2:**
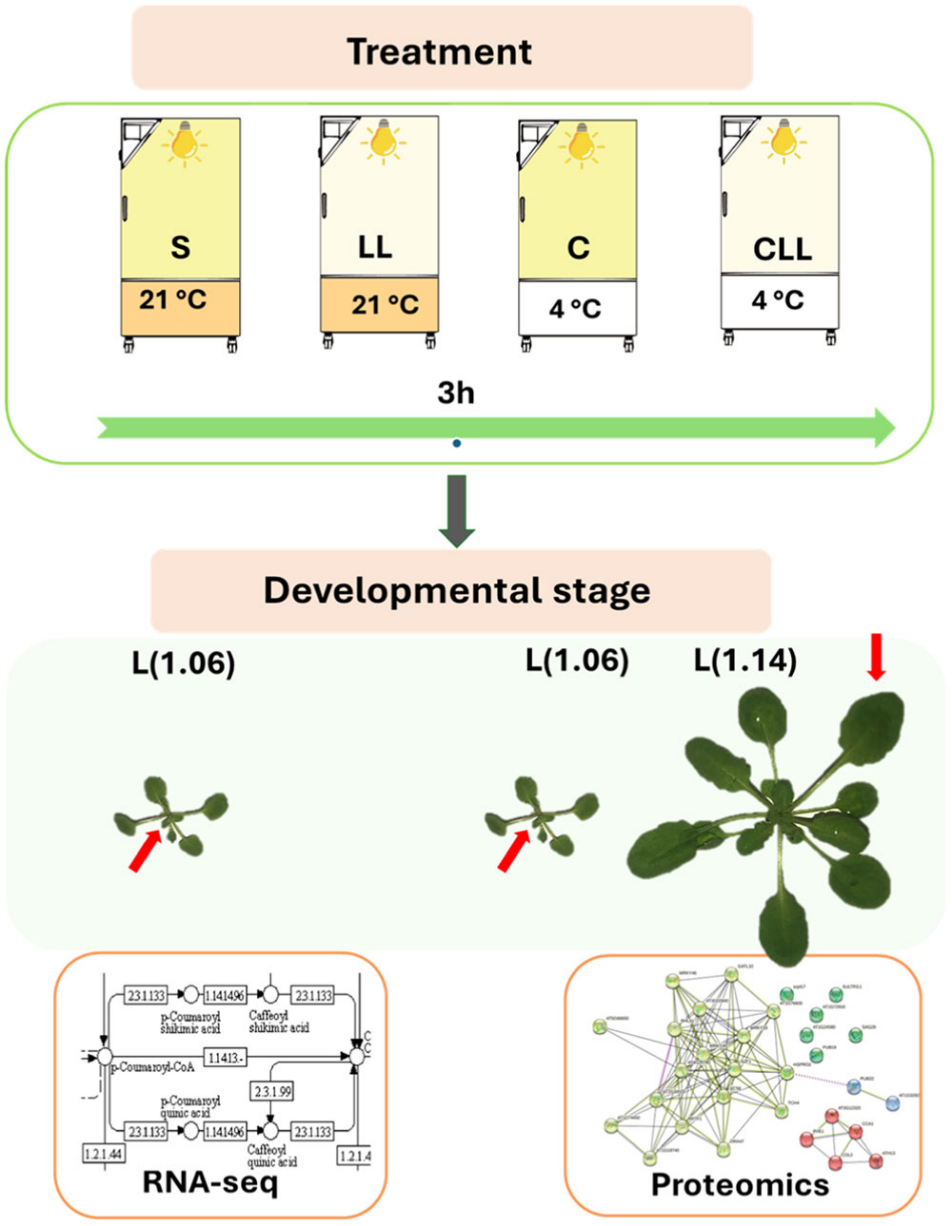
Schematic representation of experimental set‐up. *Arabidopsis thaliana* plants were cultivated at standard photosynthetic photon flux density (PPFD) until they reached developmental stage L1.06 or L1.14. Next, the plantlets were exposed for 3 h to the following four conditions: [S] 100 µmol m^−2^ s^−1^, 21°C; [LL] 20 µmol m^−2^ s^−1^, 21°C; [C] 100 µmol m^−2^ s^−1^, 4°C; [CLL] 20 µmol m^−2^ s^−1^, 4°C. Leaves n.6 (red arrows) were collected for transcriptomics and proteomic analyses. For young leaf n.6 transcriptomic and proteomic analyses of expanded leaf n.6, three biological replicates were collected, each consisting of ten leaves. For proteomic analyses of young leaf n.6, ten biological replicates were collected, each consisting of a single leaf. [Color figure can be viewed at wileyonlinelibrary.com]

**Figure 3:**
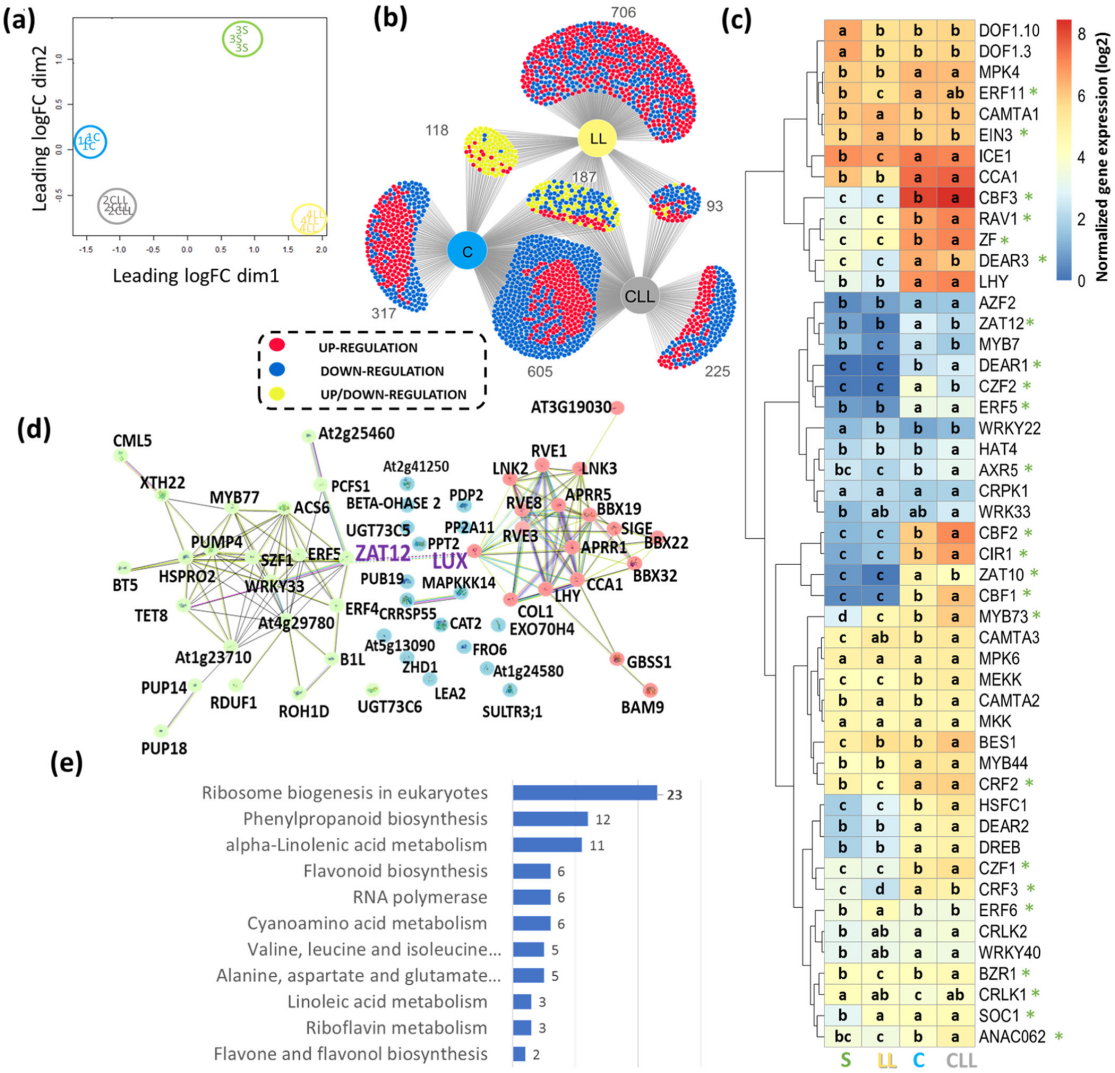
Plant's early response to cold and light treatment changes the transcriptome of the young leaf. (a) Multidimensional scaling of identified genes in RNA‐seq analysis of young leaf L1.06. (b) Comparison of identified differential gene expressions (DEGs) found in at least one treatment (FDR ≤ 0.05, absolute fold change > 2) visualized by DiVenn (Sun et al. 2019). Blue and red nodes denote downregulated and upregulated genes between different treatments (compared to control—S), respectively. Yellow nodes denote upregulation in one sample but downregulation in another. (c) Selected COR genes reportedly involved in early response to cold (Park et al. 2015; Liu et al. 2019). Significant interactions between light and cold are highlighted with an asterisk (two‐way ANOVA and Tukey post hoc test, *p* ≤ 0.05). (d) Identified DEGs that are components of circadian clock (red dots) and circadian responsive DEGs with predicted interactions (yellow dots), and missing interacting partners (blue dots). (e) Low photosynthetic photon flux density (PPFD)‐specific DEGs are enriched in ribosome biosynthesis, amino acid metabolism, and secondary metabolism pathways (*p* < 0.05). The interactions and visualisation of functional clusters were determined by STRING (Szklarczyk et al. 2019), minimum required interaction score = 0.4; S—100 µmol m^−2^ s^−1^, 21°C; LL—20 µmol m^−2^ s^−1^, 21°C; C—100 µmol m^−2^ s^−1^, 4°C; CLL—20 µmol m^−2^ s^−1^, 4°C. For details, see Supplementary Table S1 and Supplementary Figure S1. [Color figure can be viewed at wileyonlinelibrary.com]

To find molecular evidence for this hypothesis, the early transcriptional response to cold was analysed in young leaves, specifically leaf n.6, which has not finished the proliferation stage. Plantlets were exposed for 3 h to four contrasting conditions (Figure 2), including (i) standard PPFD at 21°C, 100 µmol m^−2^ s^−1^ (control; S); (ii) low‐PPFD at 21°C, 20 µmol m^−2^ s^−1^ (LL); (iii) standard PPFD at 4°C, 100 µmol m^−2^ s^−1^ (C); (iv) low‐PPFD at 4°C, 20 µmol m^−2^ s^−1^ (CLL). A young leaves L(1.06) were collected (*n* = 10, three fully independent biological replicates), and the transcriptome was analysed using RNA‐seq (Figure 3a–e, Supplementary Table S1) as described in materials and methods. In parallel, the uniformity of the targeted leaves was confirmed by the cell analyses, indicating that all collected leaves were in a similar developmental stage.

Altogether, 28 757 genes were identified and 16 549 of these passed the set criteria (present in all replicates of at least one treatment, Supplementary Table S1). Multidimensional scaling of identified genes showed a clear separation of all treatments (Figure 3a), with C and CLL treatments being separated from LL and S in the first dimension. Next, the impact of limited light (LL), cold (C), and the combination of both factors (CLL) were analysed in detail. The comparison of each of the three treatments with S revealed 2251 DEGs in total (relative FC > 2, FDR ≤ 0.05) and 1545 of these were cold‐responsive (in C and CLL) (Figure 3b). The comparison of CLL and C subsets showed a significant overlap, representing 65% and 71% of identified DEGs in C and CLL, respectively. Most of these DEGs had a similar response to cold, indicating their putative role in cold response, but the degree of their respective responses differed. Next, the data set was searched for known early cold response (COR) genes (3414 genes, Shi et al. 2017). Of these, 709 were regulated in young leaves (Supplementary Table S1), and many displayed a significant interaction of light and cold, as illustrated with the set of 49 early cold response genes (2‐way ANOVA, *p* < 0.05; Figure 3c, Supplementary Table S1), including dehydration‐responsive element‐binding proteins CBFs (CBF1, CBF2, CBF3; Figure 3c).

### 3.3 Distinct C and CLL Pathways: Impact on Photosynthesis and Phytohormone Signalling and Metabolism

As demonstrated previously (Novák et al. 2021), the CLL treatment is a combination of low‐light intensity and cold‐induced cold acclimation process through different mechanisms to that of C. Here, 317 DEGs (Figure 3b) were found only in C plants. GO analysis highlighted the impact on photosynthesis (FDR = 9.9e−05), photosynthesis‐light reaction (FDR = 0.0041), protein‐chromophore linkage (FDR = 0.013), photosynthetic electron transport chain (FDR = 0.028), response to light stimulus (FDR = 0.044), and chloroplast localisation. Detailed analyses (Table 1) showed three and six upregulated DEGs encoding core subunits of PSI and PSII, respectively. A significant upregulation was also found for genes related to photoprotective function, including Stress enhanced protein (SEP2) that binds to free chlorophyll (Ren et al. 2023), Early light‐induced proteins (ELIP1, ELIP2), that prevent accumulation of free chlorophyll by the inhibition of its synthesis, and an ATP‐dependent zinc metalloprotease (FTSH 8) involved in the removal of damaged D1 protein. The C‐induced upregulation was also found for the majority of identified plastid protein‐coding genes, with 29 out of 59 genes showing increased expression. Interestingly, cold in both C and CLL plants upregulated *CV* (Protein CHLOROPLAST VESICULATION) responsible for stress‐induced destabilisation and degradation of chloroplasts (Wang and Blumwald 2015). However, the upregulation of genes related to photosynthetic apparatus was not found in CLL plants, and only two plastid‐encoded DEGs were upregulated. The subset of C‐specific DEGs also included genes that reportedly play a role in stress tolerance and could correlate with the increased tolerance to freezing stress. These include downregulation of transcription factor *bHLH57*, which was found to increase chilling tolerance in rice plants by activating trehalose synthesis (Zhang et al. 2023) and a downregulation of a gene encoding fatty acid elongase KCS12, At2g28630).

**Table 1:** Differentially expressed genes with putative role in plant resilience to cold.

Name	AGI	Regulation C|CLL	Function	Reference
psaA	AtCg00350	↑|NR	Photosynthesis	
psaB	AtCg00340	↑|NR		
psaJ	AtCg00630	↑|NR		
psbA	AtCg00020	↑|NR	Photosynthesis	
psbB	AtCg00270	↑|NR		
psbC	AtCg00280	↑|NR		
psbD	AtCg00270	↑|NR		
psbE	AtCg00580	↑|NR		
psbK	AtCg00070	↑|NR		
SEP	At2g21970	↑|NR	Photosynthesis	Ren et al. (2023)
FTSH	At1g06430	↑|NR	Photosynthesis	Zaltsman, Ori, and Adam (2005)
ELIP	At3g22840 At4g14690	**↑**|↑	Photosynthesis	Hutin et al. (2003)
		**↑**|↑		
CV	At2g25625	↑|↑	Photosynthesis	Wang and Blumwald (2015)
bHLH57	At4g01460	↓|NR	Transcription factor	Zhang et al. (2023)
KCS12	At2g28630	↓|NR	Lipid metabolism	Kim, Go, and Suh (2018)
BZR2	At1g19350	NR|↑	Brassinosteroid singaling	Yin et al. (2002)
EXL5	At2g17230	NR|↑	Brassinosteroid response	Wang et al. (2009)
bHLH149	At1g09250	NR|↓	Negative regulator of brassinosteroid signalling	Wang et al. (2009)
FLZ17	At1g53885	NR|↑	Sugar‐repressed gene	Nietzsche et al. (2016), Jamsheer et al. (2018)
LOX4	At1g72520	NR|↑	Jasmonate biosynthesis	Yang et al. (2020)
ATHB‐6	At2g22430	NR|↑	Negative regulator of ABA	Himmelbach (2002)
CML35	At2g41410	NR|↑	Calcium signalling	
ERF060	At4g39780	NR|↓	Ethylene signalling	
XXT1	At3g62720	NR|↑	CAZyme	Culbertson et al. (2018)
GAE1	At4g30440	NR|↑	CAZyme	Mølhøj, Verma, and Reiter (2004)
BHLH137	At5g50915	NR|↑	Transcription factor	Park et al. (2021)
HAT4	At4g16780	NR|↑	Transcription factor	Schena, Lloyd, and Davis (1993)
NAC062	At3g49530	NR|↑	Transcription factor	Seo et al. (2010)
IDM2	At1g54840	NR|↓	DNA demethylation	Qian et al. (2014)
ADT3	At2g27820	NR|↓	Phenylalanine biosynthesis	Cho et al. (2007)
ZRK3	At3g57730	NR|↓	Serine/threonine‐protein kinase	
OPT5	At4g26590	NR|↓	Oligopeptide transporter	Koh et al. (2002)
PCMP	At3g12770	NR|↓	Pentatricopeptide repeat‐containing proteins	
	At4g37170	NR|↓		
NUDT17	At2g01670	NR|↓	Hydrolysis of nucleoside diphosphate derivatives	
MSD23.3	At5g46850	NR|↓	Phosphatidylinositol‐glycan biosynthesis	
RVE8	At3g09600	↑|↑	Circadian clock	Farinas and Mas (2011)
ZAT12	At5g59820	↑|↑	Circadian clock	Park et al. (2015)
BT5	At4g37610	↑|↑	Circadian clock	
LEA2	At1g02820	↑|↑	Circadian clock	
At3g19030	At3g19030	↑|↑	Circadian clock	
CCA1	At2g46830	↑|↑	Circadian clock	Alabadí et al. (2001)
LUX	At3g46640	↑|↑	Circadian clock	Hazen et al. (2005)
APRR1	At5g61380	↑|↑	Circadian clock	Matsushika et al. (2000)
PRR5	At5g24470	↑|↑	Circadian clock	Rawat et al. (2011)
LHY	At1g01060	↑|↑	Circadian clock	Mizoguchi et al. (2002)
RVE1	At5g17300	↑|↑	Circadian clock	Rawat et al. (2009)
COL1	At5g15850	↑|↑	Circadian clock	
ADO3	At1g68050	↑|↑	Circadian clock	Nelson et al. (2000)
BBX32	At3g21150	↑|NR	Circadian clock	Ravindran et al. (2021)
SIGE5	At5g24120	↑|NR	Circadian clock	Tsunoyama et al. (2004)
RVE3	At1g01520	↑|NR	Circadian clock	Kidokoro et al. (2023)
PUP18	At1g57990	↓|NR	Pathogen defence	Sheoran et al. (2016)
PCC1	At3g22231	↑|↑	Pathogen defence	Mir et al. (2013)
SNIPER1	At1g14200	↓|↓	Pathogen defence	Wu et al. (2020)
Defensin‐like protein 204	At3g05727	↑|↓	Pathogen defence	Hawamda et al. (2022)
GA2OX1	At1g78440	↑|↑	Gibberellin metabolism	Rieu et al. (2008)
PUMP6/DIC3	At5g09470	↑|↓	ROS protection (putative)	Palmieri et al. (2008)
CYP98A8	At1g74540	↓|↓	Polyamine alkaloid metabolism	Fraser and Chapple (2011)
At4g22517	At4g22517	**↑**|↓	Lipid transfer protein (putative)	
AZI1	At4g12470	↑|**↑**	Lipid transfer protein	Xu et al. (2011), Wang et al. (2016)
CYP707A3	At5g45340	↑|↑	Abscisic acid metabolism	Umezawa et al. (2006)
HSP90‐1	At5g52640	**↑**|↑	Chaperon	Kozeko (2019)
TET8	At2g23810	↑|↑	Pathogen defense	Cai et al. (2018); Jimenez‐Jimenez et al. (2019)
COL8	At1g49130	↓|**↓**	Transcription factor	Takase et al. (2011)
PLIP2	At1g02660	↑|↑	Jasmonate biosynthesis	Wang et al. (2018)

*Note:* Arrows mark regulations, and significant differences in C and CLL responses are highlighted in bold. For details, see Supplementary Table S1.

In total, 225 CLL‐specific DEGs were identified (Figure 3b). The upregulation of specific genes within the brassinosteroid signalling pathways highlighted its putative role in the CLL response, including *BRI1‐suppressor 1* (BZR2) that exhibited a noteworthy fourfold increase, and *EXL5* (a role in a brassinosteroid‐dependent regulation of growth and development). Additionally, *FLZ17*, identified as an interactor with SnRK1 (Nietzsche et al. 2016; Jamshee et al. 2018), was upregulated, suggesting its involvement in the direct control of phytohormone signalling under CLL. Moreover, the upregulation of *LOX4* and *ATHB‐6* implies an active role in jasmonic acid biosynthesis and negative regulation of ABA‐signalling pathway, respectively. Additional candidates of interests were *CML35* (potential calcium sensor), a transcription factor enhancing plant tolerance to pathogens by incorporation of cold‐mediated signalling *NAC062* (Seo et al. 2010), genes encoding carbohydrate metabolism enzymes xyloglucan 6‐xylosyltransferase 1 (XXT1) and UDP‐glucuronate 4‐epimerase 1 (GAE1), homeobox‐leucine zipper protein HAT4 and transcription factor bHLH137. In total, 92 CLL‐specific genes were downregulated. Genes with putative role in the CLL response included a negative regulator of the brassinosteroid signalling *bHLH149*, a regulator of DNA demethylation *IDM2*, *Nudix hydrolase 17* (NUDT17), *ADT3* (phenylalanine biosynthesis), *ZRK3* (Serine/threonine‐protein kinase), *OPT5* (oligopeptide transporter), genes for pentatricopeptide repeat‐containing proteins, *ERF060* (*Ethylene‐responsive transcription factor*) and *MSD23.3* (phosphatidylinositol‐glycan biosynthesis). Our analyses also pinpointed genes with previously unknown function, indicating their putative role in abiotic stress response (see Supplementary Table S1 for details).

### 3.4 Circadian Clock Genes Are Part of Early Response to Cold in Young Leaf

In *Arabidopsis*, the circadian clock is entrained by cold temperatures (Fowler, Cook, and Thomashow et al. 2005). Here, 59 DEGs associated with the circadian clock and rhythmicity were identified (Supplementary Figure S1). Approximately 45% of identified DEGs were observed in both the C and CLL plants. Five DEGs were found in all three treatments, namely *RVE8*, *ZAT12*, *BT5*, *LEA2* and *AT3G19030* (Table 1, Supplementary Tables 1). The core clock genes, including *CCA1*, *LUX*, *APRR1*, *PRR5* and *LHY*, were upregulated in both C and CLL plants. Furthermore, there was a noted upregulation of transcription factors linked to the circadian clock, such as *RVE1*, *COL1* and *ADO3*. The transcription factor ZAT12 acts downstream of CBF1 and regulates cold acclimation and stress tolerance (Park et al. 2015; Zhang et al. 2024). The STRING analysis suggested a putative interaction between ZAT12 and the cold‐upregulated circadian clock component LUX (Figure 3d). This interaction was also predicted by PEPPI (Bell et al. 2022), suggesting a previously unidentified direct link between the circadian clock and cold signalling pathways. More than 8% of circadian‐responsive DEGs were exclusively observed only in C and LL plants, and the regulation was lost in CLL. Among the candidate DEGs of interest were the zinc finger transcription factor *BBX32* (integrates light and brassinosteroid signalling; Ravindran et al. 2021), circadian clock factor *SIGE5* (mediates expression of *psbD;* Tsunoyama et al. 2004) and *RVE3* (RVE family is involved in regulating expression under temperature stress; Kidokoro et al. 2023) which were upregulated in C, downregulated in LL and not regulated in CLL plants (Supplementary Figure S1). In contrast, the transcript *PUP18* (belongs to the group of stress defense genes; Sheoran et al. 2016) encoding probable purine permease was up‐ and downregulated in LL and C plants, respectively.

### 3.5 Low PPFD Promoted Cold Stress Response of Genes Involved in Phytohormone Metabolism and Plant Immunity

The comparative analyses of C and CLL plants revealed previously unidentified cold‐responsive genes. Of particular interest were 100 DEGs that exhibited an enhanced cold stress response in CLL plants, showing an absolute fold change > 1.5 compared to C. The low‐light promoted downregulation was found for DEGs encoding defense‐related proteins PCC1 (regulates plant's pathogen defense through modulation of lipid content; Mir et al. 2013), SNIPER1 (E3 ligase involved in plant immunity response; Wu et al. 2020), Defensin‐like protein 204, an enzyme of gibberellin catabolism GA2OX1, ELIP, Mitochondrial uncoupling protein 6 (PUMP6/DIC3), Cytochrome P450 98A8 and uncharacterized gene At4g22517. A promoted upregulation in CLL plants was identified for genes encoding lipid transfer protein (AZI1; participates in systemic acquired resistance and prevents electrolyte leakage in freezing conditions; Xu et al. 2011; Wang et al. 2016), an enzyme of abscisic acid catabolism Abscisic acid 8'‐hydroxylase 3 (CYP707A3), HSP90‐1 (chaperone involved in many processes, including R gene‐mediated disease resistance), Tetraspanin‐8 TET8 (reportedly involved in cell trafficking and plant immunity; Cai et al. 2018; Jimenez‐Jimenez et al. 2019), a transcription factor COL8, FCS‐like Zinc finger 13 (FLZ13, might facilitate the interaction of SnRK complex with effector proteins; Nietzsche et al. 2016), and plastid phospholipase A1 PLIP2 that links ABA and jasmonic acid signalling (Wang et al. 2018).

### 3.6 The Analyses of the Early Proteome Response in Young Leaves Indicated the Significant Involvement of Ribosome Composition and Glutathione Metabolism in the Specific Response Observed in CLL Plants

**Figure 4:**
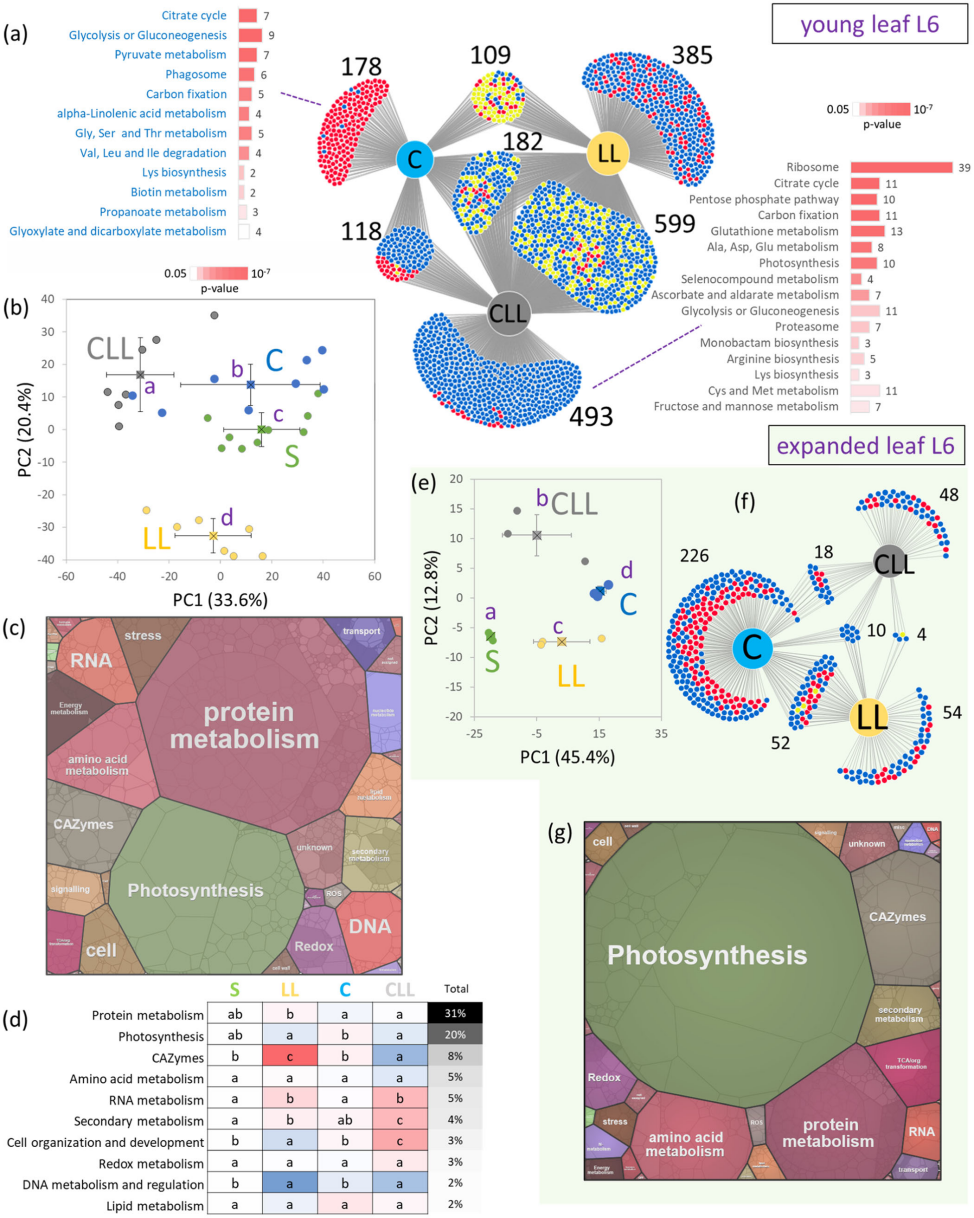
Cold response in leaf proteome. (a) DiVenn visualisation of identified differentially abundant proteins (DAPs) (*p* < 0.05, absolute fold change > 1.4) in young leaf L(1.06) and significantly enriched metabolic pathways identified by MetaboAnalyst in CLL‐ and C‐specific DAPs. (b) PCA separation based on relative protein abundances of 2064 DAPs (*p* < 0.05). (c) Proteome map illustrating the estimated protein content and the proportional distribution across diverse metabolic pathways within the young leaf of S plants, and (d) the corresponding comparison of 10 most abundant categories. (e) The PCA separation based on relative protein abundances of 487 DAPs (*p* < 0.05) identified in expanded leaf L(1.14). (f) The DiVenn visualisation of identified DAPs (*p* < 0.05, absolute fold change > 1.4), L(1.14). (g) The proteome map of expanded leaf 6 of S plants. Circles in PCAs represent individual biological replicates, squares and lines represent group means and standard deviations, respectively. Letters indicate significant differences, Kruskal–Wallis and the Conover test, *p*< 0.05). Red and blue dots in DiVenn indicate relative increase and decrease in protein abundances compared to S plants, respectively, while yellow dots represent differential responses between the comparisons. Note that ten and three biological replicates were collected for young and expanded leaf number 6, respectively. However, not all samples for young leaves were successfully extracted, and the results are based on seven (CLL, LL), eight (C), and nine (S) biological replicates. For details, see Supplementary Table S2. [Color figure can be viewed at wileyonlinelibrary.com]

To complement transcriptomic analyses, the proteome of young leaf n. 6 (developmental stage L1.06) was analysed. In total, the proteomic analyses provided identification and quantitative data for more than 5000 and 2526 Arabidopsis protein families, respectively. A significant portion of the detectable proteome showed changes in response to cold or low light intensity. Differentially abundant proteins (DAPs; adj. *p* < 0.05; absolute fold change > 1.4) represented 19.6%, 61.6% and 60.2% of estimated protein content in C, CLL and LL plants, respectively. However, the overlap in identified DAPs was formed by only 182 DAPs (Figure 4a). The PCA separated the impact of light and cold in the first and second principal components, respectively. The proteome of CLL plants was clearly separated from that of S, C and LL plants (Figure 4b), and the two‐way ANOVA confirmed an interaction between light and cold for more than 740 DAPs (adj. *p* < 0.05, Supplementary Table S2). A substantial portion of DAPs in CLL plants showed a decrease in protein abundance compared to S. However, only carbohydrate‐acting enzymes (CAZymes) and DNA metabolism exhibited a significant decrease (*p *< 0.05), while the total protein content of major categories (31% for protein metabolism and 20% for photosynthesis) remained unaltered. Moreover, there was a notable increase in the total abundance of proteins related to secondary metabolism and components of RNA metabolism and processing (Figure 4c,d).

The detailed analysis of proteome data indicated a decrease in jasmonic acid metabolism in LL and CLL plants. Specifically, the abundances of allene oxide synthase CYP74A (a key enzyme in JA biosynthesis), two plastid‐associated proteins PAPs (putative role in light/cold stress‐related jasmonate biosynthesis), and UDP‐glycosyltransferase UGT74D1 (glucosylates jasmonates) were significantly lower compared to S plants. Furthermore, cold‐induced accumulation of lipoxygenase LOX2 (JA biosynthesis), was observed exclusively in C plants. CLL plants showed a decrease in abundances for enzymes of steroid biosynthesis (SMT2 and DIM), abscisic acid biosynthesis (zeaxanthin epoxidase ZEP), and auxin metabolism (Nitrilase NIT1, amidase AMI1). In contrast, polyamine biosynthesis seemed to be stimulated in all treatments (spermidine synthase SPDSYN2), and CLL plants displayed a remarkable reduction in amine oxidase CuAOalpha2, a polyamine degradation enzyme (Table 2, Supplementary Table S2).

**Table 2:** Differentially abundant proteins with putative role in plant resilience to cold.

Name	AGI	Regulation C|CLL	Function	Reference
CYP74A	At5g42650	NR|↓	Jasmonate metabolism	Youssef et al. (2010)
PAP	At2g35490	NR|↓	Jasmonate metabolism	
	At2g35490			
UGT74D1	At2g31750	NR|↓	Jasmonate metabolism	
LOX2	At3g45140	↑|NR	Jasmonate metabolism	
SMT2	At1g20330	NR|↓	Steroid biosynthesis	
DIM	At3g19820	NR|↓	Brassinosteroid biosynthesis	Choe et al. (1999)
ZEP	At5g67030	NR|↓	Abscisic acid biosynthesis	
NIT1	At3g44310	NR|↓	Auxin metabolism	
AMI1	At1g08980	NR|↓	Auxin metabolism	
SPDSYN2	At1g70310	↑|↑	Polyamine biosynthesis	
CuAOalpha2	At1g31690	NR|↓	Polyamine degradation	
PRL1	At4g15900	NR|↓	Stress regulator	Baruah et al. (2009)
FAX3	At2g38550	NR|↓	Fatty acid transport	Li et al. (2015)
NEET	At5g51720	NR|↓	Role in development, senescence and ROS	Nechushtai et al. (2012)
uL24c	AT5G54600	NR|↓	Ribosomal protein	Romani et al. (2012)
L24‐2	AT3G53020	NR|↓	Ribosomal protein	Yao et al. (2008)
uL18y	AT5G39740	NR|↓	Ribosomal protein	Yao et al. (2008)
NOP5‐2	AT3G05060	NR|↓	Ribosome biosynthesis	
ACX3	AT1G06290	↑|↓	Lipid metabolism	
MFP2	AT3G06860	↓|↑	Lipid metabolism	Bussell et al. (2014)
REC1	At1g01320	↓|↑	Chloroplast development	Larkin et al. (2016)
UBQ12	AT1G55060	↑|↓	Protein metabolism, signalling	
L(1.14)				
HSP70	AT5G02500 AT4G37910	↑|NR	Chaperons	
		↑|NR		
SYT1	AT2G20990	↑|NR	Freezing stress tolerance	Yamazaki et al. (2009)
LOG8	AT5G11950	↑|NR	Cytokinin metabolism	
ACO4	AT1G05010	↑|NR	Ethylene metabolism	
PU1	AT5G04360	↑|NR	Starch degradation	
IPP1	AT5G16440	↑|NR	Isoprenoid metabolism	
SMT2	AT1G20330	↑|NR	Steroid biosynthesis	
OMT1	AT5G54160	↑|NR	Flavone metabolism	
PED1	At2g33150	NR|↑	Lipid metabolism	
SAD5	At2g33150	NR|↓	Lipid metabolism	
SFR1	AT3G06510	NR|↑	Lipid metabolism, critical for freezing tolerance	Moellering, Muthan, and Benning (2010)
MVD1	AT2G38700	NR|↑	Isoprenoid biosynthesis	Henry et al. (2015)

*Note:* Arrows mark regulations. For details, see Supplementary Table S2.

Proteins with a CLL‐specific response with a putative role in cold stress included fatty acid transporter FAX3 and proteins involved in stress response and stress regulation (PRL1, NEET), and the analysis of metabolic pathway enrichment in CLL‐specific DAPs showed an impact on citric acid cycle, glutathione metabolism, photosynthesis, CAZymes, amino acid metabolism and ribosomes (Figure 4a, Table 2). The proteome of CLL plants exhibited a significant decrease in abundance of eukaryotic and chloroplast ribosomal proteins. Ribosomal protein content decreased in both C and CLL plants, with CLL showing a significantly more pronounced impact, decreasing on average by 28% compared to 11% in C. In total, 127 out of 187 quantified ribosomal proteins showed a significant decrease in abundance in CLL plants, including ribosomal proteins with documented significant impact on leaf growth and development uL24c, L24‐2 and uL18y (Yao et al. 2008; Romani et al. 2012). The decrease was observed also in proteins involved in ribosome biosynthesis, including NOP5‐2. For details, see Table 2 and Supplementary Table S2.

The overlap between proteome response in C and CLL plants was substantial. In total, 301 DAPs were found in both data sets and only 11 showed contrasting response. Of particular interest were two peroxisomal enzymes involved in the fatty acid beta‐oxidation pathway (ACX3, MFP2), protein REC1 that reportedly participates in chloroplast compartment size establishment (Larkin et al. 2016), and protein Polyubiquitin UBQ12 (Table 2).

### 3.7 Early Proteome Response in Expanded Leaf n.6 Identified CLL‐Specific Alterations in Lipid Metabolism

To decipher the development‐dependent mechanisms underlying cold stress response, the proteome of expanded leaf n.6 (developmental stage L1.14) was subjected to proteome analysis and 2604 and 1982 protein families were identified and quantified, respectively. Similar to the young leaf, a distinct separation of CLL plants was observed (Figure 4e). In total, 412 DAPs were identified (*p* < 0.05, absolute fold change > 1.4), including 228, 54 and 48 specific for C, LL and C plants, respectively (Figure 4f, Supplementary Table S2). The majority of the expanded leaf n. 6 proteome represented proteins of photosynthesis, CAZymes, protein metabolism, and amino acid biosynthesis (Figure 4g). The results of the two‐way ANOVA confirmed a notable interaction between light and cold conditions, specifically impacting 174 DAPs (adj. *p*< 0.05; Supplementary Table S2). CLL plants exhibited the fewest number of DAPs, and the cold‐induced responses observed in C plants were significantly weakened and completely abolished for 68 and 213 DAPs, respectively. The DAPs that were significantly accumulated only in C plants included two HSP70 chaperons, Synaptotagmin (SYT1, critical for maintaining plasma membrane integrity during freezing stress), hormone metabolism enzymes (LOG8 and ACC oxidase ACO4), Pullulanase PU1 (starch breakdown) and three enzymes of secondary metabolism pathways (IPP1, SMT2, OMT1). While the overlap in identified DAPs was limited (Figure 4f), metabolic pathway analysis revealed that the biosynthesis of secondary metabolites, amino acid metabolism, ribosomal proteins, glutathione metabolism and CAZymes were found in all three data sets. The CLL plants showed significant enrichment in fatty acid metabolism, biosynthesis of unsaturated fatty acids, porphyrin and chlorophyll metabolism, 2‐oxocarboxlic acid metabolism, and valine, leucine and isoleucine degradation. Candidates of interest were enzymes of lipid metabolism (PED1, SAD5, SFR1) and isoprenoid biosynthesis (MVD1, mutant exhibits a significant decrease in campesterol and sitosterol content; Henry et al. 2015). Lastly, it's worth mentioning that one of the presumed cold receptors and mediators of cold acclimation, the protein ANNEXIN 1, exhibited a 1.7‐fold lower abundance in CLL plants compared to C or S. However, the regulation of this protein was only marginally missed meeting the statistically significant threshold (*p* = 0.0524).

### 3.8 Comparison of Transcriptome and Proteome Provided Validation for the Role of ROS Metabolism, Secondary Metabolism and Lipids in CLL‐Specific Response to Chilling Stress

A comparison of proteomics data from young leaves with NGS results revealed that 2447 proteins/genes were shared between the two methods, while 14 102 transcripts were undetectable by the proteome analysis. Intriguingly, 75 quantified proteins were not captured by the transcriptomics data set. A comparison of DAPs and DEGs revealed only 155 proteins/genes with overlapping profiles. Notably, only 16 and 49 displayed similar patterns in C and CLL plants, respectively. CLL‐specific responses include components of ROS metabolism (catalase 3, l‐ascorbate peroxidase 1, GSH transferases), protein At‐NEET (a role in ROS metabolism and senescence; Nechushtai et al. 2012), and the aforementioned phytohormone metabolism enzymes (amine oxidase, UDP‐glycosyltransferase UGT74D1, and auxin biosynthetic amidase). Apart from this, several enzymes involved in phenylpropanoid metabolism were also present in both data sets, alongside HSP70 proteins, nonspecific lipid transfer protein 2 (transfers phospholipids and galactolipids across membranes), and a key enzyme in the myo‐inositol biosynthesis pathway IPS1. The complete list can be found in Supplementary Table S2.

### 3.9 PLIP Family Is Involved in Cold Acclimation and Freezing Stress Tolerance

**Figure 5:**
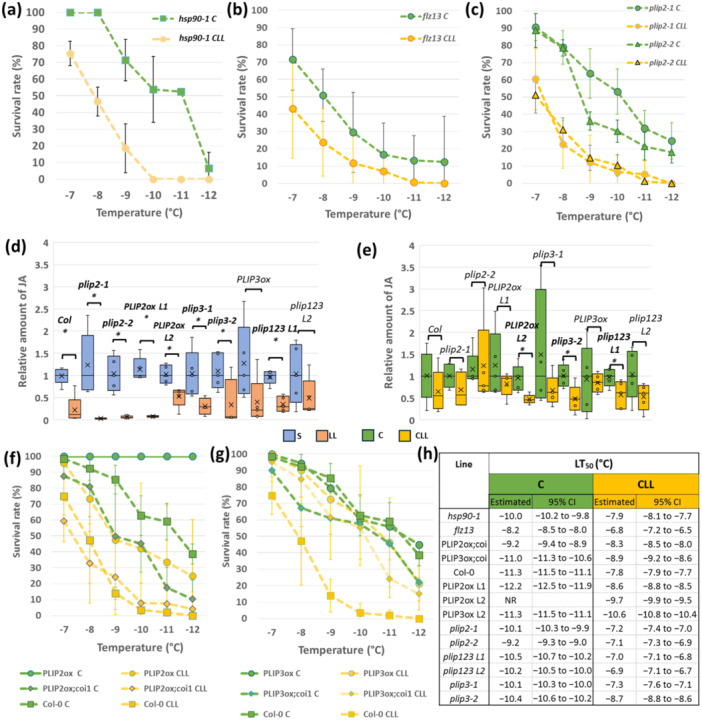
Mutations in candidate genes negatively impact freezing tolerance. (a–c) Survival rates of mutants in *HSP90*, *FLZ13 and PLIP*2. Means and standard deviation of at least three biological replicates (*n* = 25). (d, e) Relative levels of jasmonate are predominantly regulated by light. This is a simplified comparison to highlight relative differences between S and LL plants, and C and CLL plants. Absolute values were normalized to respective S and C plants. The presented data represent results of five biological replicates, each pooled from at least 60 plants. Statistically significant differences were determined using ANOVA. (f, g) Modulation of JA perception affects freezing tolerance of plants overexpressing *PLIP2* and *PLIP3*. The presented data represent results of three biological replicates (*n* > 50). (h) Calculated LT_50_ values for all mutants tested. Estimated value and 95% confidence interval (CI). [Color figure can be viewed at wileyonlinelibrary.com]

**Figure 6:**
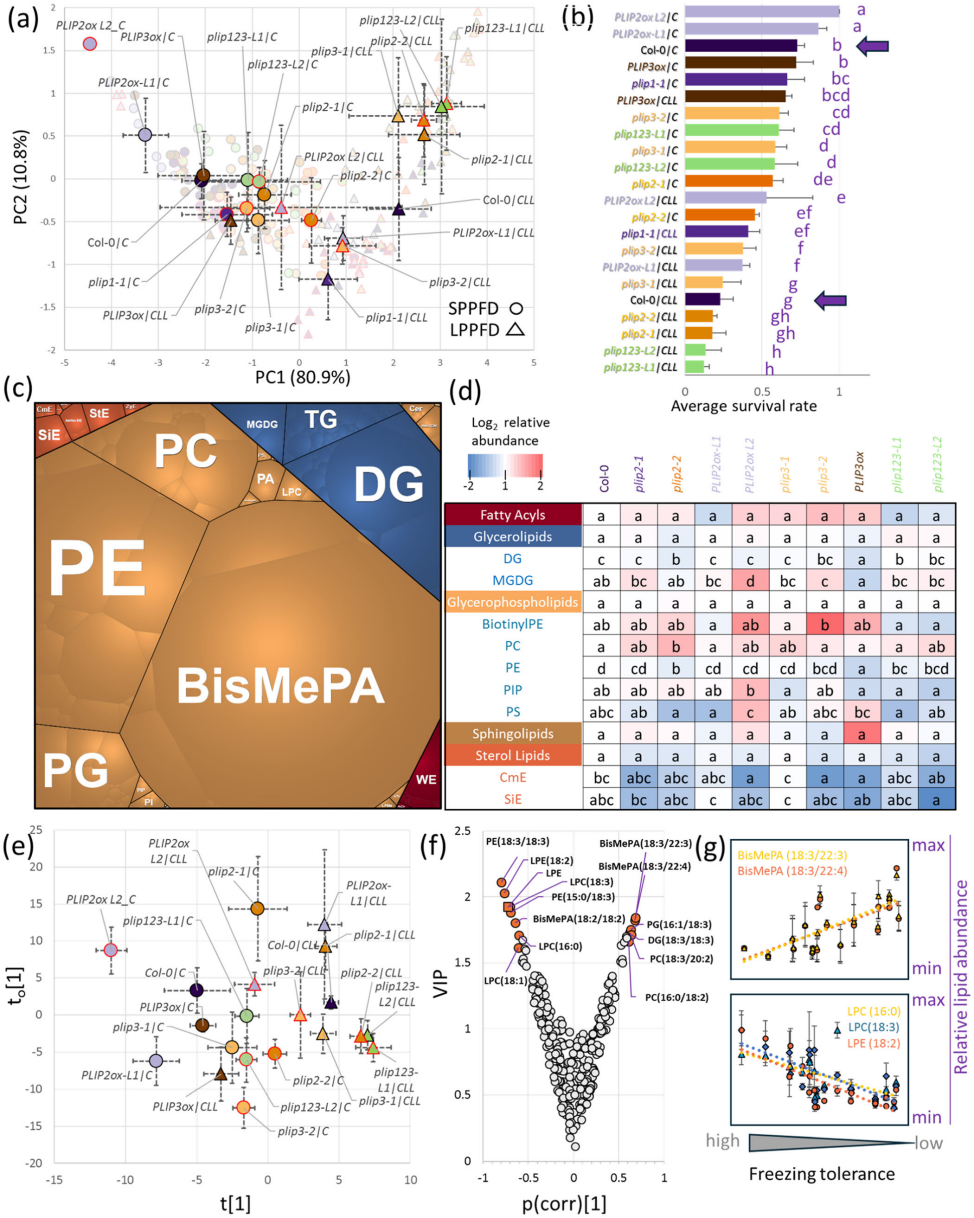
PLIP modulates lipidome and impacts freezing survival in a light‐dependent manner. (a) PCA representation of survival assay data. Means and standard deviation of at least three biological replicates (*n* = 25). (b) Mean survival rate in analysed genotypes. Bars represent means and standard deviations, the letters represent significant differences (*p* < 0.05, Kruskal–Wallis and Conover's test). Col‐0 wild‐type plants are highlighted. (c) Average lipidome composition of Col‐0 plantlet and (d) differences found in lipidome of the analysed genotypes in control samples. Letters indicate statistically significant differences (ANOVA with Tukey's HSD, *p* < 0.05). (e) Orthogonal partial least squares (OPLS) model based on freezing tolerance projection, the corresponding (f) variable importance in projection (VIP) and identified lipid compounds correlating with freezing tolerance. (g) Selected examples of relative lipid abundance changes in response to freezing stress. Results are based on at least three biological replicates. For details, see Supplementary Table S4. [Color figure can be viewed at wileyonlinelibrary.com]

**Figure 7:**
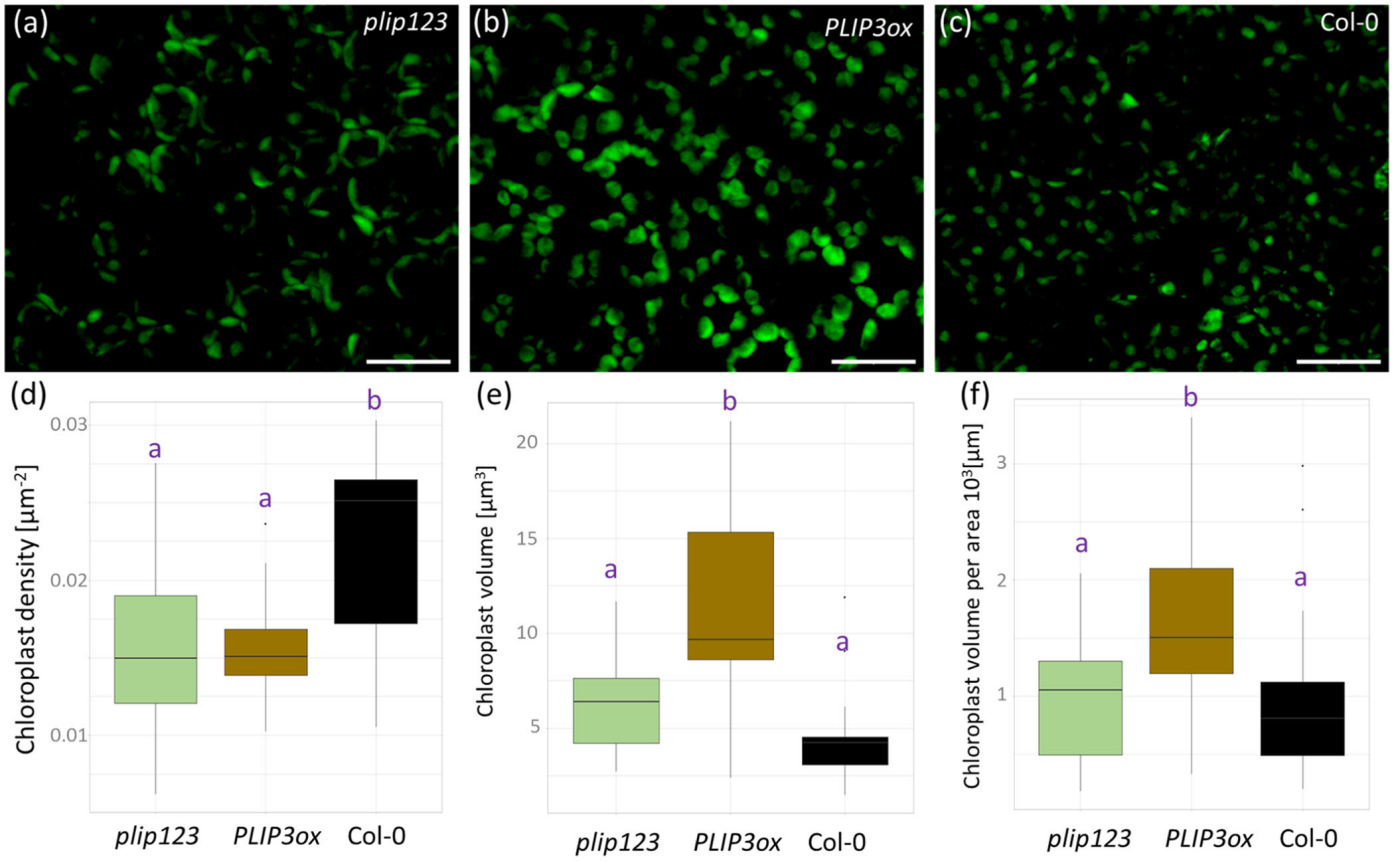
PLIP is a mediator influencing both the number and size of plastids. (a–c) Representative images of plastids in developing leaves of plip123, PLIP3ox and Col‐0 lines. Bar represents 20 µm. Box plot representations of (d) chloroplast density per area, (e) median chloroplast volume and (f) median chloroplast volume per area. Results are based on three biological replicates, each with five repeats. Letters in plots represent statistically significant differences (Kruskal–Wallis with Wilcoxon test, *p* < 0.05). [Color figure can be viewed at wileyonlinelibrary.com]

To affirm the hypothesized role of the promising candidates identified through the analyses, several Arabidopsis mutant lines showing contrasting responses between C and CLL plants were subjected to rigorous testing. The selection of mutant genotypes was based on availability, likelihood of observing functional impact from mutation, and expected gene/protein function. That encompassed mutants within *PLIP*, *FLZ13* and *HSP90‐1* where the cold‐induced upregulation was significantly higher in CLL compared to C plants. The role of these genes in cold tolerance was assessed by exposing *Arabidopsis* mutant lines to cold stress as illustrated in Figure 1a. All three loss‐of‐function mutants demonstrated notably lower resilience to freezing stress, thus affirming their involvement in the cold response mechanism (Figure 5a–c). Omics data indicated a significant role of jasmonates in contrasting survival rates between C and CLL plants. Therefore, given the role of PLIP in jasmonate metabolism, the entire gene family comprising three members was analysed in detail, and the impact on hormonome (Figure 5d,e, Supplementary Table S3), jasmonate signalling (Figure 5f,g), survival rates (Figures 5f–h and 6a,b), lipidome (Figure 6c–g), and chloroplasts (Figure 7a–f) was evaluated.

The experimental design for *PLIP* included available mutant and overexpressor lines, and the survival assay results were projected onto a PCA plot, distinctly segregating freezing stress tolerance along the first component. Moreover, it underscored a clear separation between plants that underwent acclimation under C or CLL conditions (Figure 6a). The highest freezing resilience was identified in *PLIP2* overexpressor lines acclimated under C, followed by Col‐0 and *PLIP3* overexpressor. Loss of function mutants *plip2*, *plip3* and *plip123* manifested significantly lower survival compared to Col‐0 (Figure 6b). In general, plants acclimated under low PPFD had significantly lower survival rates compared to the corresponding lines acclimated under standard PPFD, with the lowest freezing stress tolerance identified in triple mutant. Remarkably, this decline was not observed in *PLIP3* overexpressors, which exhibited comparable levels of freezing tolerance in both C and CLL plants.

### 3.10 Jasmonate Levels Do Not Appear to Be a Downstream Factor Influencing PLIP2‐mediated Resistance to Freezing

The constitutive overexpression of *PLIP2* and *PLIP3* reportedly triggers the excessive accumulation of bioactive forms of JA (Wang et al. 2018). The established correlation between this phytohormone and promoted plant resilience to low temperatures implied a potential link between JA levels and the observed resilience in *PLIP3ox* and *PLIP2ox* lines. To validate that, plants acclimated under S, LL, C and CLL conditions for 147 h were analysed, monitoring JA levels alongside other stress‐related phytohormones (Figure 5d,e, Supplementary Table S3). The results showed a higher biological variability, but the JA pool was depleted in all LL plants compared to S, and most of these changes were statistically significant (*p* < 0.05). A comparable trend was noted when comparing CLL and C plants (Figure 5d,e), except for the *PLIP3ox* and *plip2‐2* lines. The observed decrease in JA induced by low‐PPFD is in line with proteomics results that indicated an attenuation in JA metabolism in LL and CLL plants, but it does not seem to correlate with the freezing stress resilience (Figure 6a,b).

SA levels were notably reduced in response to low‐PPFD (Supplementary Figure S2, Supplementary Table S3). The impact of cold mitigated this depletion, resulting in mostly insignificant differences between C and CLL plants. Interestingly, *PLIP3ox* and *PLIP2ox‐2* exhibited increased SA levels in CLL plants compared to C. The difference did not reach statistical significance (*p* < 0.05), but it does correlate with the observed higher resilience to freezing stress. ABA was not impacted by low‐PPFD under control temperature. However, a significant decrease in ABA was observed in CLL plants compared to C plants (Supplementary Figure S2, Supplementary Table S3). While this regulation of the ABA pool doesn't explain the enhanced resilience in *PLIP* overexpressors, it does underscore a distinct acclimation mechanism under low‐PPFD.

The hormonome analysis did not show any pattern that would clearly correlate with the survival rates. However, the results of JA pool analysis could be misleading, given the differences in the absolute JA content between different lines (Supplementary Table S3). Next, the impact of *PLIP2* overexpression was assessed in lines that were crossed with mutant in a critical component of a receptor for jasmonates *CORONATINE INSENSITIVE1* (*PLIP2ox;coi1*; Wang et al. 2018). The inhibition of JA signalling diminished the impact of *PLIP2* overexpression in both C and CLL plants (Figure 5f,g). Notably, *PLIP2ox;coi1* C plants exhibited freezing tolerance similar to that of *PLIP2ox* CLL, and *PLIP2ox;coi1* CLL had a similar LT_50_ to Col‐0 CLL plants. That suggests that jasmonate signalling is integral to the PLIP2‐mediated freezing resistance. Additionally, it potentially serves as a sensor of PPFD, aligning well with previous observations (Kazan and Manners 2011).

Interestingly, the analysis of *PLIP3ox;coi1* C did not reveal reduced resistance compared to *PLIP3ox* C plants. Additionally, the survival rates of *PLIP3ox;coi1* CLL were closer to those of *PLIP3ox* CLL than the wild type Col‐0 (Figure 5g,h).

### 3.11 Shotgun Lipidome Profiling Confirmed Role of PLIPs in Response to the Combination of Light Intensity and Highlighted Putative Lipid Markers of Freezing Resistance

As PLIP is a lipid metabolism enzyme, lipidome profiles of individual lines were compared. The analysis of the leaf lipidome provided reliable identification and quantitation of more than 280 lipid compounds that represented more than 95% of the estimated lipid content. The leaf lipidome was formed predominantly by glycerophospholipids and glycerolipids, representing, on average, 77% and 18% of the Col‐0 lipidome (Figure 6c). The comparison of lipid profiles clearly separated different genotypes with significant differences in abundance (ANOVA, *p* < 0.05) found for nine subclasses (Figure 6d). To eliminate biased evaluation of lipidome response to cold in individual genotypes, lipid abundances in response to cold were normalized to the respective controls. Next, the lipidome profiles of all genotypes were analysed using the freezing tolerance projections (Figure 6a). The resulting orthogonal partial least squares (OPLS) modelling and the consecutive calculation of variable importance in projection (VIP) pinpointed a set of lipids that correlated with freezing tolerance: seven positively and six negatively (Figure 6e–g, Supplementary Table S4).

### 3.12 Plastid Number and Size Is Impacted by PLIP

Next, three lines were selected for analysis of chloroplast size and quantity (Figure 7a–f), representing control (Col‐0), line with the most attenuated resilience to cold (*plip123*), and line with a similar resilience in C and CLL plants (*PLIP3ox*). Col‐0 displayed the highest number of chloroplasts per area (Figure 7d), but the median volume of these was lower than that in *plip123* or *PLIP3ox* (Figure 6e). The total volume of chloroplast per area was significantly higher in *PLIP3ox* (Figure 6f) and could represent the factor behind the observed improved resilience in *PLIP3ox* CLL plants.

## 4 Discussion

### 4.1 Novel Cold‐Responsive Candidates Identified in Young Leaves and Missing From Arabidopsis Annotations

The physiological response of plants to environmental stimuli is influenced by their developmental stage and overall fitness (Peck and Mittler 2020). Under abiotic stress, young leaves activate protective mechanisms such as non‐photochemical quenching and the synthesis of protective compounds, which may be absent or less effective in mature leaves (Zhu et al. 2018; Rankenberg et al. 2021). Notably, significant differences in stress responses between young and mature leaves are observed under low‐temperature conditions. In this study, we investigated the early cold response in young Arabidopsis leaves grown hydroponically under contrasting PPFD and cold conditions, replicating our previous experimental setup to explore early cold‐responsive mechanisms. Our primary objective was to assess the reproducibility of our findings using a standard plate assay. To this end, we conducted parallel experiments on agar plates, which confirmed that low PPFD negatively affects the cold response, consistent with our previous hydroponic observations (Figure 1). Moreover, we demonstrated that results obtained from hydroponically grown Arabidopsis are transferable to plate‐based assays, validating the robustness of both cultivation approaches used in this study.

Despite more than two decades since the sequencing of the Arabidopsis genome and extensive collective efforts, its annotation remains incomplete. This is unsurprising, given that even the much simpler *Escherichia coli* genome still lacks functional annotation for over 15% of its protein‐coding genes (Moore et al. 2024). As of February 2025, a search of Arabidopsis gene annotations identified 441 genes associated with Gene Ontology (GO) terms related to cold responses, including GO:0009409 (response to cold, 358 genes), GO:0009631 (cold acclimation, 65 genes), GO:0010048 (vernalisation response, 18 genes), GO:0070417 (cellular response to cold, 32 genes), GO:0050826 (response to freezing, 25 genes) and GO:0071497 (cellular response to freezing, 3 genes) (Supplementary Table S5). Comparative analysis with our data sets of early cold‐responsive transcripts (Figure 3, Supplementary Table S1) and proteins (Figure 4, Supplementary Table S2) revealed a significant overlap of 85 and 63 annotated genes, respectively (Fisher's exact test, *p* < 0.001). In total, 139 genes previously annotated as cold‐responsive were identified in our omics data sets, suggesting the discovery of over 2400 novel early cold‐responsive genes and proteins (Supplementary Table S5), including candidates validated in our freezing assays (*HSP90‐1*, *FLZ13*, *PLIP2*). To further validate the roles of these putative cold‐responsive genes and proteins in cold adaptation, we analysed previously published transcriptomic data from Arabidopsis. We examined 11 data sets, encompassing six studies with seven experiments focused on early cold response: PRJNA411947 (Klepikova et al. 2019), PRJNA324514, PRJNA525452 (Yang et al. 2019), PRJNA267681 (Schlaen et al. 2015), PRJNA338072 (Pajoro et al. 2017) and PRJEB19974 (Calixto et al. 2018). An additional five studies analysing only delayed cold response were searched too: PRJNA401073, PRJNA494179 (Zhao et al. 2018), PRJNA416120 (Park et al. 2018), PRJNA445300 (Zuther et al. 2019) and PRJNA513852 (Esteve‐Bruna et al. 2020). Collectively, analysis of these studies identified over 17 600 DEGs (adj. *p* ≤ 0.05, absolute fold change > 1.5). This number likely reflects differences in the tissues and developmental stages analysed. However, at least some DEGs identified in the delayed response may represent broader alterations in growth and development, while others could result from experimental bias. Notably, the overlap of DEGs identified across multiple NGS studies is limited (Supplementary Table S5, Supplementary Figure S3), with only 760 and 1400 DEGs commonly detected in more than half of the early and delayed response analyses, respectively. Interestingly, among the 22 DEGs consistently identified across all early response studies, only one (AT1G49230) is annotated as being involved in the response to cold, while six correspond to genes or proteins with unknown functions. Of the 1379 early cold‐response DEGs identified in young leaves that lack cold‐related GO annotations, 1286 were previously reported in cold‐response NGS studies (Supplementary Figure S3), and 1086 were detected in more than three independent experiments (Supplementary Table S5). Direct comparison between early response transcripts and proteins is challenging, as protein‐level responses may be delayed by several hours, and transcriptomic and proteomic data sets offer complementary rather than identical insights into cellular processes. Nevertheless, 847 DAPs significantly affected by cold (two‐way ANOVA, adj. *p* < 0.05, Supplementary Table S2) and not annotated as cold‐related were previously identified in Arabidopsis cold‐response NGS analyses, with 373 appearing in more than three studies. Taken together, we believe that these 1086 genes and 373 proteins (a total of 1433 unique IDs) repeatedly identified in response to cold should be recognized as components of the cold acclimation process and incorporated into Arabidopsis genome annotations.

### 4.2 PLIP Plays an Integral Role in CLL‐Specific Response to Chilling Stress

The primary objective of our analyses was to elucidate the contrasting molecular processes that underlie the acclimation responses of C and CLL plants. The complementary approaches based on transcriptome and proteome analyses both highlighted putative roles for lipid metabolism and transfer. Of particular interest was the significant upregulation of *PLIP2* (AT1G02660) that encodes plastidic lipase. While the abundance of this enzyme was below the detection limits of proteome profiling, its putative impact on jasmonates biosynthesis was corroborated by evidence from both proteome and transcriptome data sets. The Arabidopsis PLIP family comprises three members, PLIP1 (At3g61680) with a well‐established role in seed oil biosynthesis, and PLIP2 and PLIP3, which are thylakoid‐membrane‐associated lipases that catalyse the initial step of jasmonic acid synthesis. Despite their shared function, PLIP2 preferentially utilizes monogalactosyldiacylglycerols (MGDG) as substrate, whereas PLIP3 prefers phosphatidylglycerol (PG) as its substrate (Wang et al. 2018). None of these enzymes has been annotated with a cold response function (Gene ontology annotations, 02/2025; Supplementary Table S5). However, data mining of available NGS studies identified *PLIP2* in three previous analyses of early cold response (median log_2_FC = 1.6) and both *PLIP2* and *PLIP3* in late cold response (Supplementary Table S5). To our knowledge, the role of these enzymes in cold acclimation or freezing tolerance has not been experimentally validated. The experiment with mutant and overexpressor lines confirmed that PLIP2 abundance positively correlates with freezing stress resilience (Figure 6a,b). The overexpressor lines displayed the highest survival rates, whereas the *plip2* mutant lines displayed the second lowest rates, marginally better than those observed in the triple mutant *plip123*. Moreover, the increased survival rates observed in *plip1* and *plip3* mutants compared to Col‐0 in CLL plants might be associated with a potential compensatory mechanism and the accumulation of PLIP2 in these mutant variants. The experiment additionally validated that the absence of PLIP has a notably more pronounced impact under low PPFD conditions. While the highest decline in survival rates compared to Col‐0 was 21% in C plants (*plip2*), it escalated to 10% in CLL plants (*plip123*).

### 4.3 The Comparison of the Proteome Response Between Young and Expanded Leaves Hints at a Scenario Where Expanded Leaves Might Be Killed to Sustain the Growth of Young Ones

As expected, the proteome profile of the expanded leaf 6 differed significantly from its earlier developmental stage at 1.06 (Figure 4c,g). There was a notable increase in the allocation of resources towards photosynthesis and CAZymes, while the processes associated with protein metabolism and RNA processing showed a distinct reduction. The comparison of quantified proteins revealed an overlap of 1514 proteins between both data sets, while 1012 were unique to data set 1.06 and 469 to data set 1.14 (Supplementary Table S2). The response to cold was significantly attenuated in 1.14 plants, indicating that altering composition of these leaves was not a priority. The observed differences could correlate with the difference in plant size and the higher capacity to compensate stress response. However, it aligns with the established sink‐source relationship, where actively growing leaves act as a sink for resources, while maintaining them comes at the expense of mature leaves (Chang and Zhu 2017). Furthermore, it corroborates our findings that younger leaves exhibited greater resilience to cold stress compared to mature ones (Figure 1c). Interestingly, 16 DAPs discovered in 1.14 CLL plants exhibited a similar trend to those identified in 1.06 CLL plants, including an enzyme of fatty acid biosynthesis (beta‐ketoacyl‐[acyl‐carrier‐protein] synthase III; AT1G62640, ↑), flavonoid biosynthesis (flavonol 7‐O‐rhamnosyltransferase; AT1G06000, ↑), a cis‐acting element 14‐3‐3‐like protein GF14 nu (AT3G02520, ↑), beta‐amylase 3 (AT4G17090, ↓) that mediates accumulation of maltose upon freezing stress (Kaplan and Guy 2005), and chloroplastic protein WHY1 (AT1G14410, ↓) that maintains plastid genome stability (Maréchal et al. 2009).

### 4.4 Plastid Maintenance Could Represent the Major Pathway That Promotes Freezing Resilience in C Plants

One of the primary effects caused by low temperatures is the inhibition of enzyme activity, particularly within the Calvin cycle, the main consumer of light energy, leading to cascading effects on associated metabolic pathways and redox status (Ruelland et al. 2009; Teh et al. 2023). During cold acclimation, young leaves exhibit a more efficient re‐establishment of photostasis, facilitated by enhanced activation of metabolic enzymes (Strand et al. 1999). This mechanism mitigates over‐reduction of the photosynthetic electron transport chain, thereby limiting the formation of reactive oxygen species that could exacerbate low‐temperature stress‐induced cellular damage. Major portion of DEGs and DAPs indicated significant alterations in photosynthesis and related metabolic pathways in C and CLL plants (Figure 4A, Table 1, Supplementary Tables S1 and S2). A notable upregulation of plastidic genes in C plants, might suggest that CLL plants are struggling to manage damage to their plastids. Alternatively, it could indicate that an inadequate PPFD isn't providing enough energy to sustain fully functional photosystems. In a long term, that could explain lower resilience of CLL plants. That likely corresponds to the outcomes of the plastid volume analysis (Figure 7a–f). The observed lower quantity of chloroplasts in *plip123* mutant, in comparison to Col‐0, seems to coincide with its status as the least resilient line. In parallel, *PLIP3ox*, the line displaying minimal PPFD impact on freezing resilience, demonstrated a notably larger total chloroplast volume than Col‐0. It should be noted that plastids are also subject to degradation through autophagy, a process that serves both nutrient recycling and quality control purposes (Izumi et al. 2015). A notable rise in polyamine production in C and CLL plants (Table S2) might suggest NO‐mediated autophagy (Minibayeva et al. 2023), where plants with a greater number/size of plastids could potentially benefit more.

### 4.5 Lipid Composition Correlates With Freezing Resilience

PLIP enzymes demonstrate a broad substrate specificity in vitro. Nevertheless, PLIP1 and PLIP3 exhibit a preference for cleaving chloroplastic phosphatidylglycerols, while PLIP2 primarily targets monogalactosyldiacylglycerol (MGDG) as its substrate (Wang, Froehlich, et al. 2017). Beyond the previously documented influence on jasmonate biosynthesis attributed to PLIP2 and PLIP3, supported by our hormonal profiling (Supplementary Table S3), the impact of PLIPs extends to the modulation of lipidome composition (Figure 6c–g), and it is possible that this modulation could be the major reason for the observed freezing stress resilience. Lipid composition remodelling is crucial for cold tolerance (Gao et al. 2024) and here, the OPLS identified 13 compounds of interest that correlated with freezing resilience (Figure 6f), including *lyso*‐phosphatidylethanolamines (LPE), *lyso‐*phosphatidylcholines (LPC), and Bis‐methyl phosphatidic acids (BisMePA). Both LPE and LPC (abundances positively correlate with resilience) were found in previous analyses of cold stress (e.g., Liu et al. 2022). Interestingly, LPE is an inhibitor of phospholipase D that mediates plant responses to stresses, and its inhibition promotes freezing tolerance of both non‐acclimated and cold‐acclimated plants (Rajashekar et al. 2006). The inhibition of phospholipase D additionally suppresses the production of phosphatidic acid, a trend that aligns with the observed negative correlation between BisMePA and both LPE levels and freezing resilience (Figure 6g).

### 4.6 PLIP Family Shows No Clear Association With the Global Distribution of Arabidopsis Accessions

Given the observed impact of *PLIP* on cold acclimation and freezing resilience, we investigated its potential role in adaptation using the 1001 Genomes Project (Weigel and Mott 2009). Specifically, we examined associations between *PLIP* mutations and expected cold resilience in Arabidopsis accessions. Only one accession carried a high‐impact mutation in *PLIP1* (Mnz‐0) and *PLIP2* (Ga‐0), while no such mutations were identified for *PLIP3*. This suggests the functional importance of these genes, which is unsurprising given their presumed essential role in plastid formation, as indicated in our study. The search for moderate‐impact mutations yielded more results, with the combined high & moderate mutation data set encompassing 207, 954 and 678 accessions for *PLIP1*, *PLIP2* and *PLIP3*, respectively. However, when comparing accession distribution with expected climatic conditions, statistically significant deviations from the estimated distribution were observed only for *PLIP1* (Supplementary Figure S4). Overall, these findings suggest that the *PLIP* family plays a crucial role in Arabidopsis growth, and mutations in available accessions do not indicate any clear associations with adaptation.

## 5 Conclusion

Our study presents an omics‐driven discovery of factors involved in cold acclimation and enhanced freezing resilience in young leaves. Through integrative transcriptomic and proteomic analyses, we identified over 1400 DEGs and 1100 DAPs in response to cold stress and demonstrated that light intensity significantly influences their regulation and accumulation. Despite extensive prior research on early cold responses and cold stress adaptation, a substantial proportion of the identified genes and proteins had not been previously annotated as cold‐responsive. However, data mining in publicly available resources revealed a statistically significant overlap with previous NGS‐based analyses, and the metabolic processes enriched in our data set align with those previously associated with cold stress responses. To validate our omics findings, we selected three promising candidates (PLIP, FLZ13 and HSP90‐1) that exhibited significantly distinct responses to cold and cold stress under low PPFD. Using a freezing resilience assay, we confirmed their role in cold stress adaptation. Given the established role of the PLIP family in plastid lipid metabolism, we further characterized all three Arabidopsis isoforms in detail. Our findings provide compelling evidence that PLIP‐mediated freezing resilience is associated with the modulation of chloroplast number and size, as well as the accumulation of lipids previously linked to cold resistance. Together, these results offer novel mechanistic insights into the role of PLIP in cold acclimation and our collected omics data present a valuable resource for future investigations into cold stress adaptation.

## Conflicts of Interest

The authors declare no conflicts of interest.

## Supporting information

Supporting Figure S1. Supporting figure for transcriptomics: regulation of circadian‐responsive genes, interaction networks, and functional enrichment of Low PPFD‐specific DEGs.
Supporting Figure S2. Phytohormone analyses.
Supporting Figure S3. Comparison of identified early response DEGs with results found in previous NGS analyses.
Supporting Figure S4. *PLIP* family mutations in Arabidopsis accessions.

Supporting Table S1. Supporting data for transcriptomics.

Supporting Table S2. Supporting data for proteomics.

Supporting Table S3. Supporting data for hormonomics.

Supporting Table S4. Supporting data for lipidomics.

Supporting Table S5. Analysis of publicly available data on cold response.

## Data Availability

The data that support the findings of this study are available in supporting information and in the following data repository: https://www.ncbi.nlm.nih.gov/geo/, accession GSE278942; http://www.ebi.ac.uk/pride, accession PXD050271.
