# Supplementary material for: Light Quantity Impacts Early Response to Cold and Cold Acclimation in Young Leaves of Arabidopsis
Source: Plant Cell Environ. 2025 Mar 27;48(7):5030–52. doi: 10.1111/pce.15481 (PMC12131964; doi:10.1111/pce.15481)
Supplement: Supplementary file 1 — Supporting Figure S1. Supporting figure for transcriptomics: regulation of circadian‐responsive genes, interaction networks, and functional enrichment of Low PPFD‐specific DEGs. Supporting Figure S2. Phytohormone analyses. Supporting Figure S3. Comparison of identified early response DEGs with results found in previous NGS analyses. Supporting Figure S4. PLIP family mutations in Arabidopsis accessions. [file PCE-48-5030-s006.docx]

1.
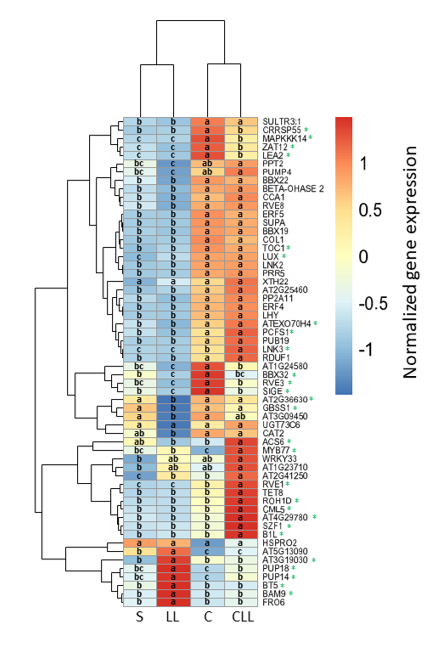
 **(b)**


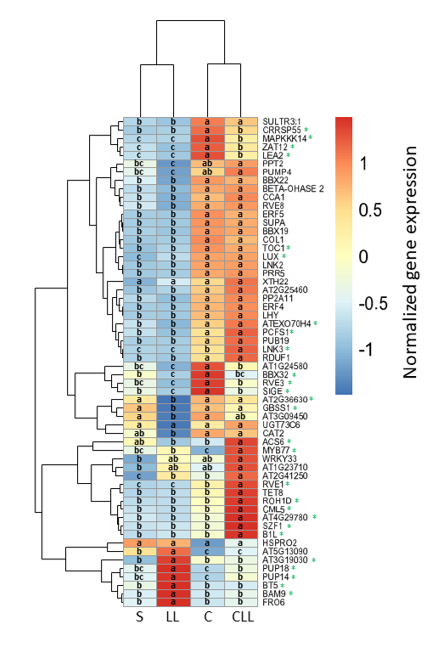


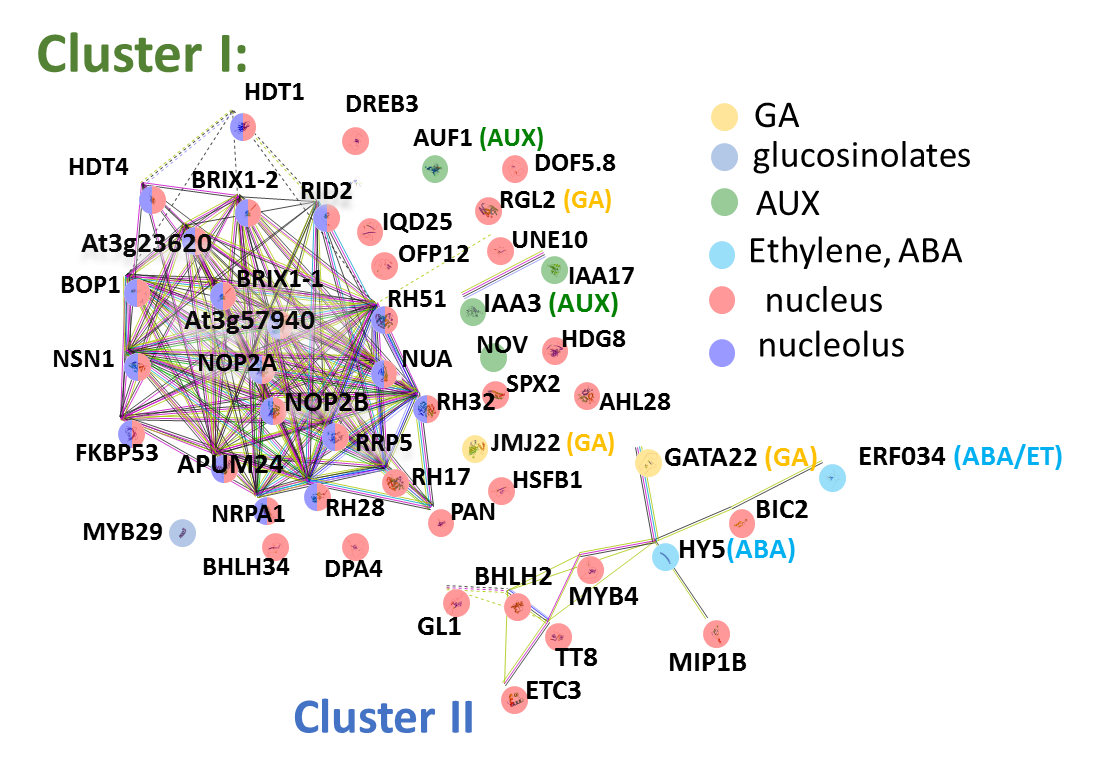


**Figure S1:** **Supplementary figure for transcriptomics: regulation of circadian-responsive genes, interaction networks, and functional enrichment of Low PPFD-specific DEGs.** **(a)** Combination of C- and low-PPFD impacted regulation of circadian responsive genes. DEGs found in RNA-seq analyses after 3-hour treatment. Significant interactions between low-PPFD and cold (p-value<0.05) are highlighted with an asterisk. The results of two-way ANOVA and Tukey post hoc test (p-value<0.05). **(b)** Low PPFD-specific DEGs are enriched in signaling and glucosinolate metabolism. The interactions and visualization of functional clusters were determined by STRING (Szklarczyk *et al.* 2019), minimum required interaction score = 0.4. For details, see Supplementary Tables S1.


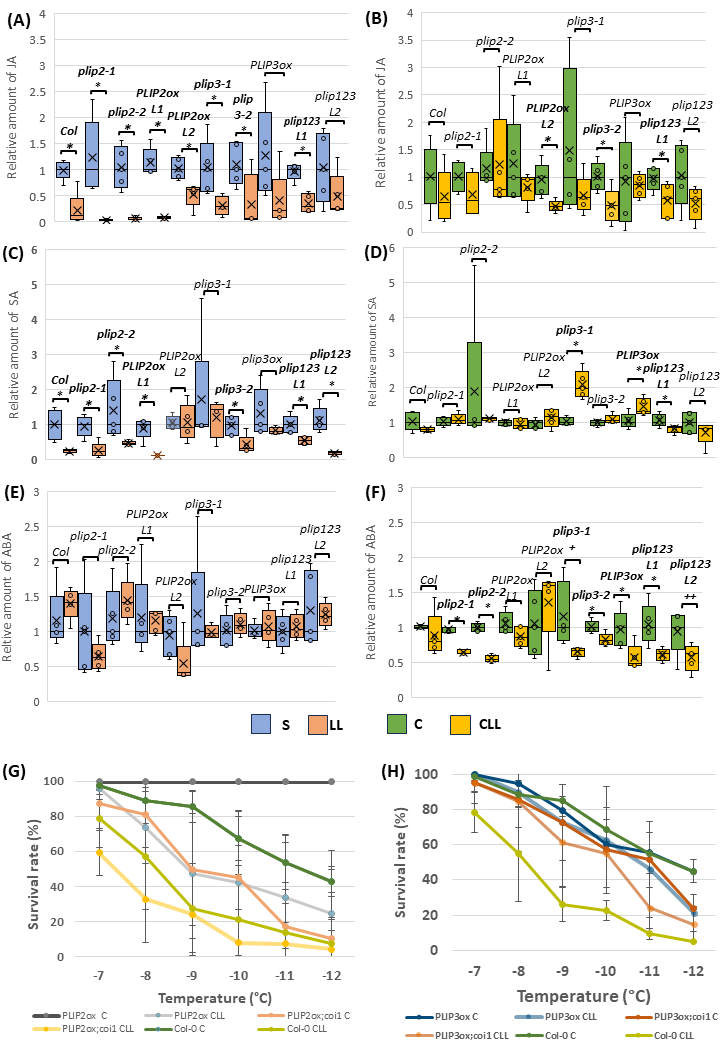
**Figure S2**. **Relative levels of plant hormones are predominantly regulated by light.** A simplified comparison to highlight relative differences between S and LL plants (A, C, E), and C and CLL plants (B, D, F). Absolut values were normalized to respective S and C plants. The experiment design and abbreviations are identical to those in Figure 6A. The presented data represent results of five biological replicates, each pooled from at least 60 plants. For details, see Supplementary Tables S3.


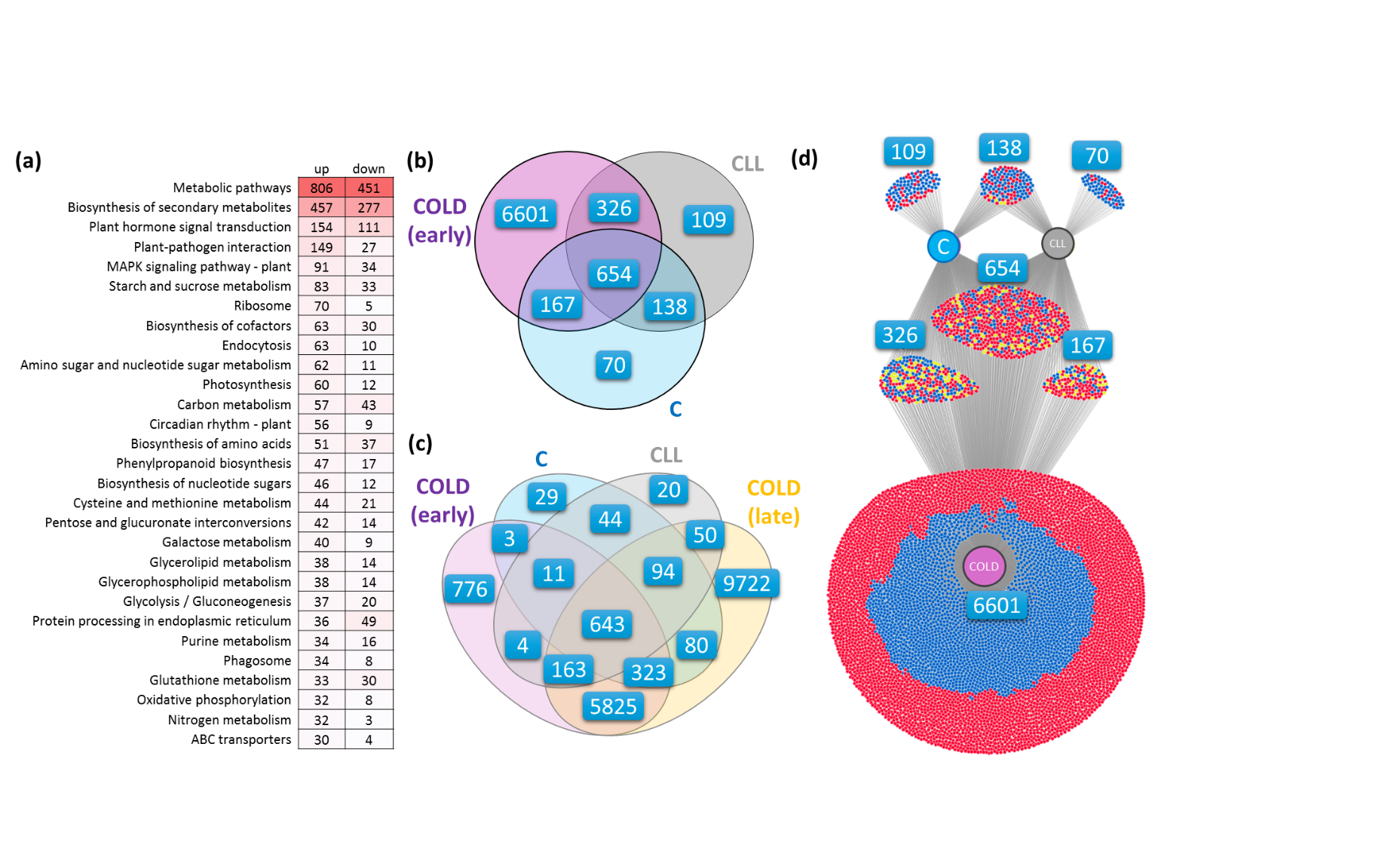


**Figure S3. Comparison of early response DEGs**. **(a)** Major metabolic pathways commonly identified in previous NGS studies investigating the early response to cold stress. Data are based on six studies (PRJNA411947, PRJNA324514, PRJNA525452, PRJNA267681, PRJNA338072, PRJEB19974). Numbers indicate the total count of differentially expressed genes (DEGs) encoding enzymes associated with each pathway across all studies. The up/down designation represents the median expression change (upregulation or downregulation) across the datasets; **(b-c)** Overlap of DEGs identified across multiple NGS studies on early cold response and delayed response showing the number of shared and unique DEGs among the datasets; **(d)** Comparison of identified DEGs found in at least one treatment (FDR ≤ 0.05, absolute fold change >2, Supplementary Table S1) and previous analyses (FDR ≤ 0.05, median absolute fold change >1.5, Supplementary Table S5) visualized by DiVenn. Blue and red nodes denote downregulated and upregulated genes between different treatments, respectively. Yellow nodes denote upregulation in one sample but downregulation in another. COLD – median regulation of identified DEGs in previous studies; C – 100 µmol.m^-2^.s^-1^, 4 °C; CLL - 20 µmol.m^-2^.s^-1^, 4 °C; For details, see **Supplementary Table S5**.


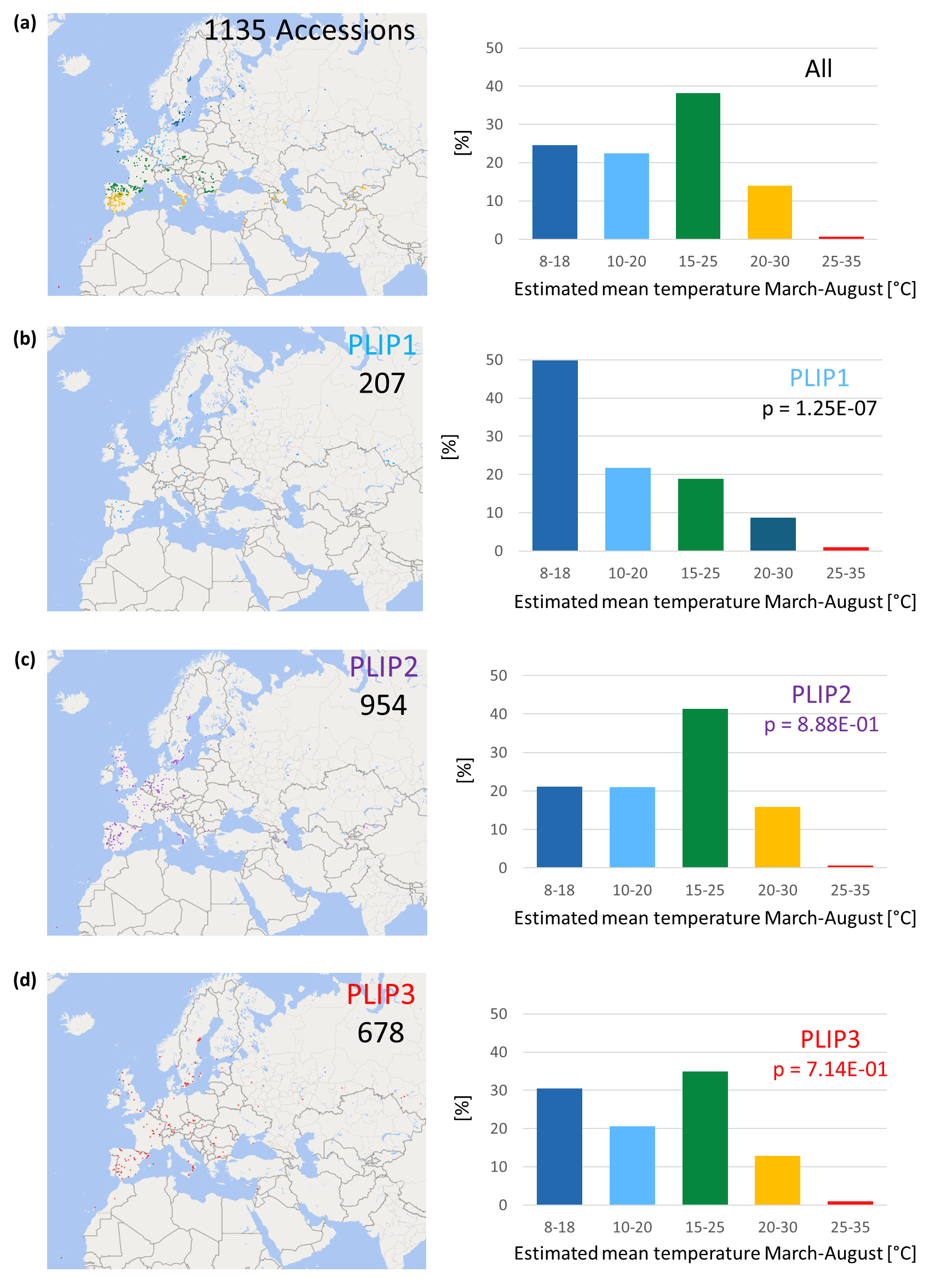


**Figure S4. *PLIP* family mutations in Arabidopsis accessions. (a)** Overview of 1,135 Arabidopsis accessions distributed based on estimated mean temperature during the March–August period; **(b–d)** Accessions carrying high- or moderate-impact mutations in PLIP genes. Data were retrieved from the 1001 Genomes Project (https://1001genomes.org/). The map focuses on regions containing the majority of observed accessions and was generated using MS Excel. The reported p-values correspond to the results of the chi-squared test.
